# Machine learning models for smart grid stability prediction: a comparative analysis

**DOI:** 10.1038/s41598-026-47385-x

**Published:** 2026-04-17

**Authors:** Ahmed M. Ali, Omar K. Dawoud, Osama A. Ghoneim, Mohamed Abdel-Basset

**Affiliations:** 1 Faculty of Computers and Informatics, Zagazig University, Zagazig, Sharqiyah 44519 Egypt; 2 Faculty of Computers and Informatics, Tanta University, Tanta, Egypt

**Keywords:** Machine learning, Smart grid, Renewable energy, Optimization, Data preprocessing, Energy science and technology, Engineering, Mathematics and computing

## Abstract

As renewable energy sources and variable demand increase, maintaining the stability of smart grids (SGs) helps guarantee that electricity systems continue to operate effectively and uninterrupted. More intelligent solutions are required since traditional monitoring methods frequently examine the initial indications of instability. This study proposes a machine learning (ML) methodology for classification and prediction of SG stability to obtain effectiveness in system operations and increase reliability. Fourteen ML models are used in this study for classification and prediction tasks. These ML models are tested under eight evaluation metrics. This study uses the features of engineering and selection to improve the model’s performance and accuracy and reduce the dimensionality. Different feature selection methods are used, such as filter, wrapper, and embedded methods. We used two hyperparameter optimization methods, such as Bayesian optimization (tree-structured Parzen estimator, TPE) and metaheuristic optimization (grey wolf optimizer, GWO), to improve ML performance. The results show that light gradient boosting machine achieves near-perfect predictive performance under both optimization strategies, with the TPE-based model reaching 99.95% accuracy and the GWO-based model reaching 99.90%. We use different explainable AI methods to ensure the model is trustworthy. This study improves SG resilience and supports energy efficiency.

## Introduction

The need for energy, specifically electrical energy, has increased recently due to the world’s population expansion, economic activity, and increasing growth^1^. Consequently, electrical energy, which may be generated from sources including hydroelectricity, wind, solar, fossil fuel, thermal, and even nuclear energy, becomes an essential energy source ^2^. Increased generation capacity and the significance of electricity management are driven by the rising demand for power. The energy grid, an enormous and complicated structure that connects power providers and consumers, operates under this management^3^. The transmission lines, distribution circuits, substations that control voltage, and power-generating plants make up the grid.These components work together to ensure a continuous and reliable flow of electricity. However, the massive quantity of demand exceeds the centralized structure of conventional electric grids, which can lead to overloading, reduced power quality, and the need for additional power plants. Furthermore, complex forecasting techniques for forecasting intermittent power outages or calculating their causes, analysis, reaction time prediction, and storage needs evaluation are absent from traditional networks^4^.

These outdated systems are under a lot of strain due to the world’s population expansion, which causes mechanical switches to slow down reaction times and decrease efficiency, monitoring, and control^5^. Variable weather, rising energy demand, component failures, and heavy reliance on fossil fuels are additional stresses on conventional power systems. In a world where energy demand is always increasing, a shift toward smarter, decentralized, and resilient grid alternatives is required to achieve sustainable energy security^6^. Innovations in smart grid (SG) technology, an important means of addressing the growing energy needs of modern society while enhancing everyone’s quality of life, are quickly changing the old design of the electrical infrastructure. All things considered, the SG systems improve the safety, effectiveness, and dependability of power systems by orders of magnitude by facilitating two-way communications between power providers and consumers. Using real-time energy consumption data, the suggested architecture for dynamic energy exchange, which in turn helps consumers make educated decisions about their use^7^. Similar approaches have been applied to energy consumption prediction in smart buildings^8^.

Data on customer demand is gathered and continuously examined in this SG, assisting operators in observing availability levels and use trends^9^. To enable optimal energy consumption, the user can receive the current pricing information through such an analysis. The integration of these contemporary communication technologies with SGs enables more efficient power generation^10^. Grid stability is a significant problem, though, because the system relies on precise operational coordination and real-time response. Although SGs make it easier to overcome most of the drawbacks of conventional power systems, they also present several socioeconomic and technological challenges^11^.

On a technical level, these include cybersecurity risks associated with grid digitalization, difficulties using appropriate energy storage devices, and insufficient regulatory frameworks. Furthermore, future energy storage demands, which would increase with a greater reliance on renewable energy sources like solar and wind, cannot be supported by the present grid infrastructures^12^. The increasing use of electric cars, which necessitates appropriate vehicle-to-grid power flow control strategies, further complicates energy management. Other issues like power distribution management, system stability maintenance, and power oscillation administration are brought on by the enormous amount of data produced by the vast number of interconnected devices. These include socioeconomic difficulties such as policy concerns, raising awareness, engaging stakeholders, and requiring significant financial outlays for the implementation of SG. A safe, robust, and sustainable energy future will be ensured by overcoming these many obstacles^13^.

With previously unattainable levels of efficiency, dependability, and sustainability, SG’s use of machine learning (ML) and artificial intelligence (AI) technologies has brought new light to the production, distribution, and consumption processes^14,15^. The enormous data volumes gathered from various SG components are analyzed using ML models, which provide real-time decision-making and predictive analytics. To dynamically optimize utilities’ production and distribution, for example, it can estimate patterns of energy demand based on past consumption data, weather, and even societal trends^16^. This flexibility will be especially useful when combining naturally variable energy sources like the sun and wind. ML models forecast output variability and dynamically adjust grid operation to improve stability.

Additionally, by allowing protection maintenance and reducing downtime through advanced problem detection and diagnosis, ML enhances grid management ^17^. These technologies can detect potential problems in the grid infrastructure before they become critical. by using techniques like anomaly detection. Additionally, ML improves cybersecurity by identifying anomalous patterns that can point to a potential attack on the grid system in the future. While it comes to energy storage, AI can maximize efficient energy consumption by optimizing battery filling and emptying cycles with the goal of storing energy while it is abundant and releasing it during periods of high demand. ML integration enhances customer interaction as well. Customers may control energy use more carefully and cut expenses by using smart meters, which can track consumer consumption trends and provide timely, individualized recommendations. Demand response systems, which would encourage customers to modify their usage during peak hours to lessen the burden on the grid, would also be feasible on these platforms^18^.

**Fig. 1:**
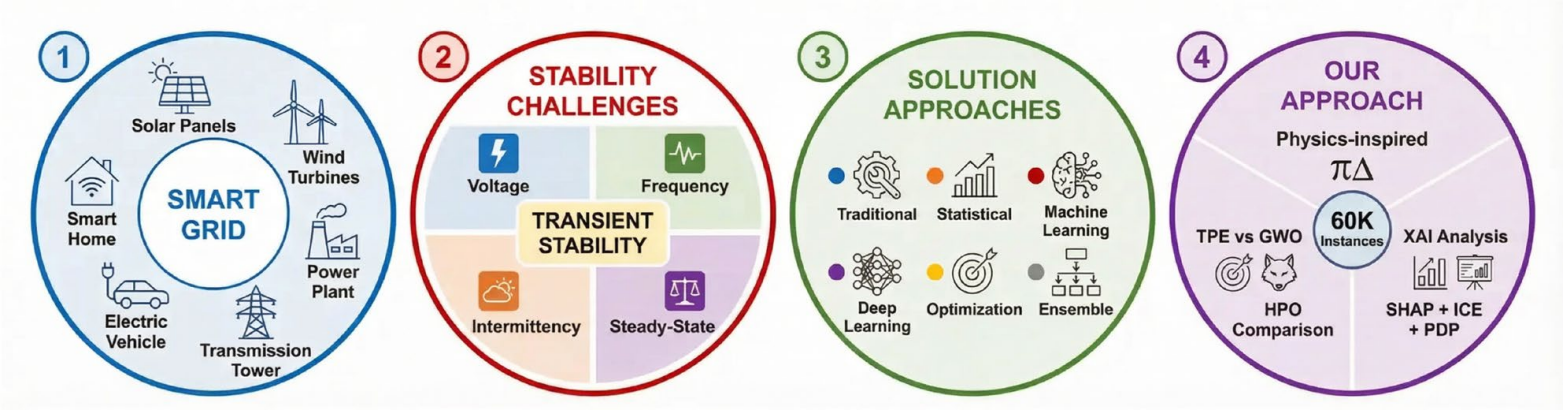
Integration of ML with SG stability prediction framework.

The SG architecture integrating ML methods is shown in Fig. 1. Power production units, transmission lines, distribution networks, and clients are only a few of the segments that make up the whole SG, as shown in Fig. 1. The grid performs better in terms of process monitoring, control, and optimization when different ML methods are integrated into it at different functions. Smart meters and advanced sensors continually collect data about energy use and system performance. The gathered data is subjected to more sophisticated ML algorithms, which enable pattern analysis to forecast future energy needs and problem identification to satisfy the grid’s final power distribution^19^. This ML-driven design effectively improves the SG’s competency, dependability, and sustainability by allowing for improved energy resource management with less downtime and condition adaptation^20^.

As the complexity of ML models in power systems increases, the need for interpretability and transparency becomes critical. Explainable artificial intelligence (XAI) techniques enable grid operators to understand and validate model decisions, which is essential for trustworthy deployment in critical infrastructure^21^. By combining advanced ML algorithms with XAI methods such as SHAP, PDP, and ICE plots, operators can gain confidence in model predictions while maintaining high accuracy and computational efficiency.

The main contributions of this study are organized as follows: The current work presents a crucial issue in SG management, accurately forecasting the grid’s stability condition, which is essential for guaranteeing dependable power transmission and system resilience. Effective stability forecasting is essential for minimizing system failures and improving operational performance as modern power systems increasingly incorporate a variety of energy sources, storage technologies, and demand-response mechanisms. Therefore, this study adds to the literature by providing a thorough assessment of 14 ML models from five groups such as: gradient boosting (extreme gradient boosting (XGBoost), light gradient boosting machine (LightGBM), categorical boosting (CatBoost), gradient boosting), tree ensembles (random forest, AdaBoost), support vector machines (SVC with RBF and Linear kernels), and linear/probabilistic models (logistic regression, stochastic gradient descent classifier (SGD), K-nearest neighbors (KNN), linear discriminant analysis (LDA), quadratic discriminant analysis (QDA), Naive Bayes). Even though the individual components of the proposed framework, namely exploratory data analysis (EDA), preprocessing, feature engineering, model training, and XAI, are well-represented in the field of ML, the contribution of this research lies in the integration of these components and their application to the field of SG stability assessment. The proposed framework integrates two different approaches to hyperparameter optimization (HPO), namely Bayesian TPE and metaheuristic GWO, along with physics-inspired features to improve the performance of the predictive model while ensuring the overall interpretability of the results, as opposed to the traditional approaches of applying these individual components of the proposed framework, as presented in the literature.

Additionally, the study evaluates the models used to classify SG stability states using the augmented SG stability dataset^22^. To ensure that each model operates under optimal conditions, two HPO strategies are investigated: Bayesian optimization using the tree-structured Parzen estimator (TPE) and a metaheuristic approach based on the grey wolf optimizer (GWO). This comparison provides insight into the effectiveness of different optimization paradigms for SG stability prediction.

In addition, this study applies several data preprocessing steps to enhance the representation of the dataset, including feature engineering and feature selection. In particular, physics-inspired feature transformations are introduced based on the response delay and elasticity parameters of the DSGC model in order to encode stability-relevant relationships in the learning representation. To further improve model transparency, multiple XAI techniques are employed, including SHapley Additive exPlanations (SHAP), partial dependence plots (PDP), and individual conditional expectation (ICE) plots. Finally, the proposed framework is compared with previous studies on the same dataset in terms of predictive performance, computational efficiency, and interpretability.

The rest of this study is organized as follows: Section "Literature review" reviews studies of ML models related to SG stability and prediction to increase performance and accuracy. Section "Methodology" shows the ML methodology used in this study, with a description of the dataset and steps of the data preprocessing to improve ML performance. Section "Results and discussion" shows the results and discussion of the ML models. Also, shows the comparative analysis results between different HPO methods and other related ML studies to show the effectiveness of the proposed approach. Section "Conclusion, limitations, future works" shows the conclusions of this study and future direction related to ML and SG stability.

## Literature review

A review of several SG research solutions is presented in this part, giving a comprehensive overview of SG technologies and highlighting areas for future development. Tiwari et al.^23^ verified the use of the augmented dataset, achieving 98% accuracy and 99% AUC with SVM. These initial studies indicated that the problem is addressable by standard ML techniques. Once they were sure that it was possible, the researchers turned their attention to making it more accurate. This made them explore ensemble-based methods. Hassan et al.^24^ compared standard classifiers with ensemble methods and found that stacking several models got 97.77% accuracy. Allal et al.^34^ employed an alternative approach to address class imbalance, integrating K-means SMOTE with CatBoost to achieve 99.6% accuracy. Other ensemble variations also saw similar improvements. In a different approach, Alaerjan et al.^25^ looked at how voting and Dempster-Shafer fusion methods compared, finding that a simple voting scheme could reach 99.8% accuracy. Seeking efficiency for edge computing, Ahakonye et al.^26^ developed a lightweight 1D CNN tailored for low-power devices; he successfully maintained a 99.79% accuracy rate despite the hardware constraints. Lakshmanarao et al.^27^ tested a hybrid model that combined an SVM with an artificial neural network, resulting in an accuracy of 98.92%. Taken together, these various efforts demonstrate that accuracy for ensemble and fusion methods has largely converged above the 98% mark.

More recently, hybrid deep learning (DL) approaches have emerged as a powerful paradigm for classification tasks across diverse domains^28^. These methods combine multiple neural network architectures with attention mechanisms to enhance feature extraction and model interpretability^29^. Such hybrid approaches are particularly valuable for critical infrastructure applications where both high accuracy and explainability are essential.

In the past, many researchers used default or very slightly tweaked hyperparameters. This made it unclear how much systematic modification might really improve performance. Breviglieri et al.^30^ looked at this by testing different optimizers like Adam, Nadam, and SGD They tested these and were able to get 99.62% accuracy with DL. Abu Al-Haija et al.^31^ were even more direct in their approach. They used an optimized SVM and achieved 99.93% accuracy after 30 iterations of tuning. Their predictions required only 4.17 microseconds, demonstrating that high precision could be achieved without sacrificing real-time speed. Despite these strong results, the ideal optimization strategy for grid stability remained a point of debate. Wang et al.^12^ sought a more systematic answer by comparing GridSearchCV against Bayesian optimization. He demonstrated that Bayesian methods generally outperformed standard grid searches, securing 96.00% accuracy with an SVM. In a different direction, Karim et al.^32^ looked toward nature-inspired algorithms. He used the Guide-WWPA method to tune LSTM hyperparameters with SMOTE for data balancing, reaching 99.7% accuracy and proving that metaheuristic approaches are indeed competitive in this space. While Wang focused on Bayesian methods and Karim looked at metaheuristics, no one has directly compared these two fundamentally different philosophies using the same data and conditions. Furthermore, they rarely reported the computational cost involved, an oversight that matters significantly for power grids where models must be retrained periodically as new data flows in.

Recent advances in HPO frameworks have further improved the efficiency of model tuning. Optuna, a modern HPO framework, has been successfully applied to hybrid ML models for fault detection in power transmission systems^28^. These developments demonstrate that systematic HPO, combined with hybrid model architectures, can achieve superior performance while maintaining computational efficiency—a critical requirement for real-time grid applications.

As model accuracy matured, the research focus shifted toward a more critical gap: interpretability. While high accuracy was needed, it was not enough for actual deployment if grid operators cannot decipher the reasoning behind a predicted instability. They could not simply trust the model if they did not know how it worked. Ucar^33^ was among the first to tackle this by applying SHAP and PDP to a Gradient Boosting model. Although he achieved 99.6% accuracy, these tools primarily highlight global patterns, showing which features matter on average rather than explaining specific, individual predictions. Furthermore, he noted that PDP might be unreliable when features interact heavily. In an effort to bridge this gap, Allal et al.^34^ used LIME alongside SHAP to extract local insights. However, LIME is often unstable, as minor changes to the input data can trigger wildly different explanations. More recently, Cifci^35^ offered a more thorough investigation by pairing SHAP with ICE plots. By testing ten different architectures, he maintained a 96.2% accuracy rate while successfully providing both global and local perspectives. His work demonstrated that a combined XAI strategy produces a far more complete picture than any single method used alone.

The importance of explainability extends beyond grid stability to broader critical infrastructure applications. Recent work on explainable LSTM-based fault diagnosis systems^36^ demonstrates that advanced DL architectures can be made interpretable through systematic XAI approaches, even in high-stakes domains such as aircraft engine fault diagnosis. These methodological advances are directly applicable to power system stability prediction, where operators must understand and trust model decisions.

Despite these advances, three important gaps remain. First, no study has directly compared Bayesian and metaheuristic optimization methods on the same problem. Wang et al.^12^ showed that Bayesian optimization outperformed a basic grid search, while Karim et al.^32^ demonstrated that metaheuristic methods can also achieve strong performance. However, it is still unclear which approach performs better under the same conditions for this task. In addition, the computational cost of these optimization strategies is rarely reported, even though this factor is important for power grid applications where models may need to be retrained as new data becomes available.

Second, most prior studies used the dataset variables in their original form^22^. Although XAI techniques can identify which variables are important, the features themselves were not designed to explicitly represent relationships related to system responsiveness. Power system stability is influenced by the interaction between response delays and corrective actions^37^. Motivated by this perspective, features can be engineered to capture such interactions in a more structured way. In this work, we explore this idea by introducing physics-inspired feature transformations derived from the response delay and elasticity parameters of the DSGC model. The objective is not to construct a full physical model, but to enrich the feature representation with stability-relevant relationships that are not explicitly encoded in the original variables.

Third, recent studies addressing interpretability have primarily applied XAI methods to models trained on the original dataset features. For example, Cifci^35^ provided a comprehensive interpretability analysis using SHAP and ICE plots, but the analysis was still based on the raw variables. By incorporating physics-inspired features, the resulting explanations can potentially reflect structured relationships between system variables rather than isolated measurements. This may help produce interpretations that are more meaningful in the context of grid stability analysis.

## Methodology

**Fig. 2:**
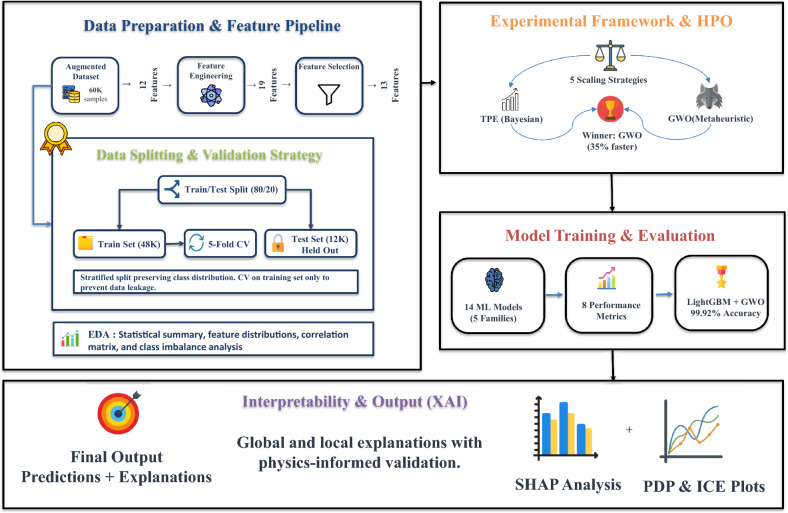
Comprehensive methodology overview.

This section shows the proposed approach include five steps to obtain higher accuracy and performance (Fig. 2). The initial phase in the pipeline is EDA, followed by data preprocessing and physics-inspired feature engineering. We then compare feature scaling methods and HPO strategies across 14 classification models. After training and evaluation, we apply several XAI techniques to interpret model predictions and verify that the learned patterns reflect actual physical mechanisms rather than data artifacts.

### Experimental configuration

Table 1 provides a comprehensive summary of the experimental configuration used in this study.

**Table 1:** Experimental configuration summary.

Category	Parameter	Value
Data	Train-test split	80%/20%
	Cross-validation	Stratified 5-fold
	Random seed	42
	Total samples	60,000
Features	Original features	12
	Engineered features	7
	Selection method	Consensus voting ($\ge 3/6$)
	Final features	13
Optimization	Methods	TPE, GWO
	Trials per model	50
	Models evaluated	14
	Performance metric	Mean CV accuracy
Evaluation	Metrics	8 (Acc, Prec, Rec, F1, MCC, AUC, LL, Kappa)
	Confidence intervals (CI)	95% bootstrap (5000)
	Test set size	12,000
	CV folds	5
Environment	OS	Windows 11
	Python	3.13.2
	RAM	15.73 GB
	CPU cores	8 logical, 4 physical

All hyperparameter search ranges are specified in the tables below. Complete code and reproduction instructions are available in the GitHub repository (see Code availability section).

### Dataset description

We use the augmented UCI SG stability dataset^22^, which contains 60,000 samples generated from simulations of a four-node star network. The dataset is derived from the decentralized smart grid control (DSGC) model introduced by Schäfer et al.^38^. This topology represents a simplified decentralized SG architecture consisting of one central producer connected to three consumer nodes.

The dataset contains twelve predictive variables describing the operational state of the grid. These variables capture three main aspects of the system: the response delay of grid agents, the power exchanged within the network, and the sensitivity of agents to price signals. Table 2 summarizes the original input variables and their ranges.

The objective of the learning task is to predict the binary target variable stabf, which indicates whether the grid operates in a stable or unstable state.

**Table 2:** Original features of the SG stability dataset.

Feature	Node role	Description	Range/unit
tau1	Producer	Reaction time of producer to price signals	[0.5, 10.0] s
tau2	Consumer 1	Reaction time of consumer 1	[0.5, 10.0] s
tau3	Consumer 2	Reaction time of consumer 2	[0.5, 10.0] s
tau4	Consumer 3	Reaction time of consumer 3	[0.5, 10.0] s
p1	Producer	Power produced (computed to satisfy power balance)	Per unit (p.u.)
p2	Consumer 1	Power consumed by consumer 1	$[-2.0,-0.5]$ p.u.
p3	Consumer 2	Power consumed by consumer 2	$[-2.0,-0.5]$ p.u.
p4	Consumer 3	Power consumed by consumer 3	$[-2.0,-0.5]$ p.u.
g1	Producer	Price elasticity coefficient of producer	[0.05, 1.0] s$^{-1}$
g2	Consumer 1	Price elasticity coefficient of consumer 1	[0.05, 1.0] s$^{-1}$
g3	Consumer 2	Price elasticity coefficient of consumer 2	[0.05, 1.0] s$^{-1}$
g4	Consumer 3	Price elasticity coefficient of consumer 3	[0.05, 1.0] s$^{-1}$

### Feature engineering and feature selection

We used a two-step process consisting of feature engineering followed by feature selection. Feature engineering was first applied to construct additional variables derived deterministically from the original dataset attributes. Because these transformations do not rely on dataset-level statistics, they can be safely computed prior to the train–test split. Feature selection was then performed exclusively on the training dataset after the split in order to avoid information leakage.

#### Feature engineering

In many ML studies, input variables are treated as independent predictors without explicitly considering relationships suggested by the underlying system dynamics. In power systems, however, stability behavior is governed by interactions between response delays, corrective actions, and power imbalance. To incorporate this domain knowledge into the learning process, we introduce several engineered features inspired by principles from power system stability theory, as detailed in Table 3

The dynamic behavior of synchronous generators is commonly described by the swing equation^37,39^:

1 $$\begin{aligned} M \frac{d^2\delta }{dt^2} + D \frac{d\delta }{dt} = P_m - P_e \end{aligned}$$

where *M* is the inertia constant, *D* represents damping effects, $\delta$ denotes the rotor angle, and $P_m-P_e$ corresponds to the power imbalance driving the system dynamics. The swing equation provides a reduced-order representation of electromechanical behavior and remains widely used for analyzing transient and small-signal stability in modern power systems^40^. From a dynamical perspective, system stability depends strongly on the balance between inertia-related response delays and damping mechanisms that counteract oscillatory deviations.

The dataset used in this work originates from the DSGC model described earlier. Within this framework, each node is characterized by two key parameters: a reaction time $\tau _i$, representing the delay in response to price signals, and a price elasticity coefficient $g_i$, representing the sensitivity of power consumption or production to price changes. These parameters influence how quickly agents respond to deviations in the system and how strongly they adjust their power behavior.

Although these variables are not direct measurements of generator inertia or damping coefficients, their interaction affects the dynamic responsiveness of nodes within the DSGC framework. The relationship between response delay and corrective sensitivity therefore reflect mechanisms that are conceptually analogous to inertia–damping interactions in classical stability models.

Based on this observation, we construct seven additional variables designed to capture these interactions. Node-level interaction terms $\tau _i g_i$ represent the coupling between response delay and control elasticity at each node, while aggregated variables summarize the overall responsiveness of the network. These engineered features expand the original feature set from 12 to 19 variables.

**Table 3:** Engineered features.

Feature	Description and rationale
tau_mean	Mean reaction time $\left( \frac{1}{4}\sum _{i=1}^{4}\tau _i\right)$ representing the overall response latency of grid agents.
g_sum	Sum of price elasticities $\left( \sum _{i=1}^{4} g_i\right)$ representing the aggregate responsiveness of agents to price signals.
tau1_g1	Interaction between reaction delay and price elasticity for the producer node $(\tau _1 g_1)$.
tau2_g2	Interaction between reaction delay and price elasticity for consumer node 1 $(\tau _2 g_2)$.
tau3_g3	Interaction between reaction delay and price elasticity for consumer node 2 $(\tau _3 g_3)$.
tau4_g4	Interaction between reaction delay and price elasticity for consumer node 3 $(\tau _4 g_4)$.
g_tau_ratio	Ratio between aggregate elasticity and mean latency $\left( \frac{g\_sum}{\tau _{mean}}\right)$ capturing the balance between system responsiveness and delay effects.

The engineered features are deterministic transformations derived directly from the original variables and do not rely on any dataset-level statistics. Therefore, they do not introduce information leakage and can be safely computed prior to the train–test split.

To examine whether these engineered variables artificially simplify the classification task, we analyze the separability of the feature space in Section  "Feature space separability analysis"

#### Feature selection

To identify the most relevant predictors from the expanded 19-feature set, we implemented a consensus-based feature selection strategy combining filter, wrapper, and embedded methods. This multi-method approach reduces the bias associated with relying on a single technique and improves the robustness of the selected feature subset^41^.

To avoid information leakage, the dataset was first split into training and test sets. All feature selection procedures were performed exclusively on the training data, while the test set remained completely unseen throughout the pipeline.

To assess the robustness of the selected feature subset, we conducted an additional validation in which the feature selection procedure was repeated independently within each fold of a repeated stratified cross-validation scheme (5$\times$5 folds). This analysis was used to evaluate the stability of the selected features and to verify that performing feature selection once on the training set does not introduce measurable bias.

The feature selection pipeline consisted of three stages. First, four filter methods were applied to evaluate features independently of the learning model. A variance threshold was used to eliminate quasi-constant variables. Next, a correlation filter removed redundant predictors where the Pearson correlation coefficient exceeded 0.95. To capture more complex dependencies between features and the target variable, we additionally applied mutual information, which detects nonlinear relationships, and an ANOVA F-test, which measures linear statistical significance.

Second, a wrapper method was applied using recursive feature elimination (RFE) with a RandomForest estimator. RFE iteratively removes the least informative variables while retraining the model, allowing feature interactions to influence the ranking. This provides a more realistic assessment of feature importance compared with purely univariate filters.

Third, an embedded method was used by training a RandomForest classifier and extracting Gini importance scores as part of the model training process. Embedded methods integrate feature evaluation directly into the learning algorithm and therefore capture complex non-linear dependencies.

The outputs of the six selection techniques were then combined using a majority-voting scheme. A feature was retained if it was selected by at least three of the six methods, corresponding to a majority-consensus rule. This ensures that a feature is supported by multiple selection techniques while avoiding overly aggressive pruning of potentially complementary predictors.

### Model preparation

Model preparation aims to ensure that input features are transformed in a manner consistent with the assumptions and optimization requirements of different ML algorithms. In this study, preprocessing is treated as a model-dependent design component, with particular emphasis on systematically selecting feature scaling strategies under a leakage-free cross-validation framework.

#### Feature scaling

Given the heterogeneous ranges and distributional characteristics of the SG features, multiple scaling strategies were evaluated rather than adopting a single global preprocessing scheme. Specifically, we considered five transformations from scikit-learn: StandardScaler (z-score normalization), MinMaxScaler (range [0,1]), RobustScaler (median and interquartile range), PowerTransformer (Yeo–Johnson transformation), and QuantileTransformer (distribution mapping), in addition to using the raw features without scaling as a baseline.

The choice of scaling was treated as a model-dependent design decision. From a theoretical perspective, feature scaling influences model behavior through its impact on optimization dynamics, distance computations, and distributional assumptions. Linear and gradient-based models depend on feature magnitude for stable convergence and effective regularization. Kernel and distance-based methods rely on Euclidean distances and are therefore sensitive to feature scale, while probabilistic models (e.g., LDA, QDA) assume approximately Gaussian-distributed inputs. In contrast, tree-based models partition the feature space using threshold-based splits and are largely invariant to monotonic transformations of the input variables^42^.

Accordingly, each scaling strategy was evaluated separately for every model using 5-fold stratified cross-validation on the training set. To prevent information leakage, scaling was applied within the cross-validation loop using a pipeline, ensuring that transformation parameters were learned exclusively from the training folds. For each model–scaler combination, both the mean and standard deviation of the validation accuracy across folds were computed. The scaler yielding the highest mean validation performance was selected for that model and subsequently used in the HPO stage, where it was incorporated into the cross-validation pipeline to ensure leakage-free model tuning. This model-specific approach avoids imposing a uniform preprocessing scheme and enables a fair and controlled comparison of algorithms under preprocessing conditions aligned with their learning mechanisms, in line with recent empirical findings on scaling sensitivity across model families^42^.

### ML models

To obtain a comprehensive benchmark, we evaluated fourteen classification models representing several widely used algorithm families. The selected models include gradient boosting methods (XGBoost^43^, LightGBM^44^, CatBoost^45^, GradientBoosting), tree ensembles (RandomForest, AdaBoost), support vector machines (SVC with RBF and linear kernels), instance-based learning (KNN), and linear or probabilistic models (logistic regression, SGD, LDA, QDA, and Naive Bayes).

This selection was designed to cover diverse learning paradigms with different inductive biases and decision boundary characteristics. Linear and probabilistic models provide interpretable baselines, instance-based and kernel methods capture nonlinear relationships, while ensemble tree methods are widely recognized for their strong performance on structured tabular datasets^46^. Including models from these complementary families enables a systematic comparison of algorithm behavior on the SG stability dataset and reduces the risk of drawing conclusions based on a single model class. The final set of fourteen models therefore reflects a balance between methodological diversity and algorithms commonly used in both ML and SG prediction studies.

### Hyperparameter optimization

HPO is crucial for ML performance, as hyperparameter selections can substantially affect predictive performance^47^. In general, HPO involves balancing exploration of diverse regions of the search space with exploitation of configurations that have already shown promising performance.

In this study, HPO was integrated into the overall experimental pipeline. All optimization experiments were conducted using pipelines that incorporated the previously selected, model-specific feature scaling strategy, thereby ensuring consistent preprocessing and preventing information leakage. Under this setting, two complementary HPO strategies were implemented and compared: TPE as a Bayesian optimization approach, and GWO as a population-based metaheuristic approach. Both methods were evaluated under identical preprocessing and validation conditions to ensure a fair comparison.

#### Tree-structured Parzen estimator (TPE)

TPE is a sequential model-based optimization technique that constructs a probabilistic model of the objective function to guide the search for optimal hyperparameters^48^. Implemented using the Optuna framework^49^, TPE diverges from traditional Bayesian methods that model *p*(*y*|*x*) (the probability of performance *y* given hyperparameters *x*). Instead, TPE models *p*(*x*|*y*), where *x* denotes a hyperparameter configuration and *y* corresponds to the associated validation performance, by partitioning the observed configurations into two groups, a “good” group and a “bad” group, based on a predefined performance threshold $y^*$ (e.g., a quantile of observed validation scores).

The core of TPE’s selection strategy is to maximize the ratio of the likelihoods of promising and non-promising configurations, as defined by the expected improvement (EI) criterion:

2 $$\begin{aligned} \text {EI}(x) \propto \frac{l(x)}{g(x)} \end{aligned}$$

By selecting the hyperparameter set *x* that maximizes this ratio, TPE prioritizes configurations that are more probable in the distribution of good results, *l*(*x*), and less probable in the distribution of bad results, *g*(*x*). This mechanism allows the search to progressively focus on more promising regions of the hyperparameter space.

In this study, TPE is applied within the proposed experimental pipeline using model-specific preprocessing and stratified cross-validation. Algorithm 1 summarizes the experiment-specific optimization procedure adopted in this study.

**Algorithm 1:**
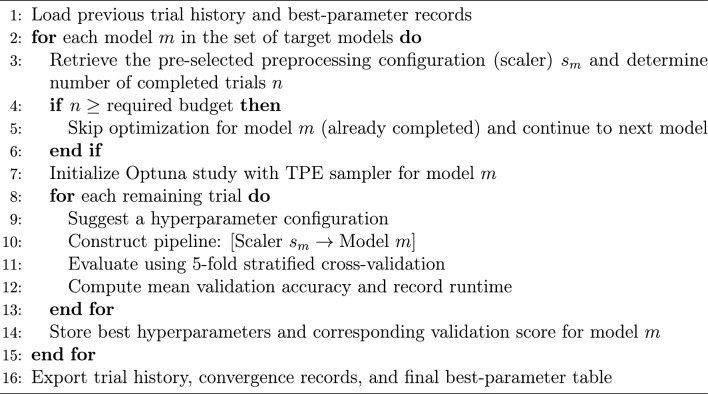
Experiment-specific TPE optimization procedure

For TPE, each candidate hyperparameter configuration was evaluated using 5-fold stratified cross-validation on the training set, and the objective function was defined as the mean validation accuracy across the five folds.

#### Grey wolf optimizer (GWO)

GWO is a nature-inspired metaheuristic algorithm that models the social hierarchy and cooperative hunting behavior of grey wolves^50^. The algorithm maintains a population of candidate solutions, where each “wolf” represents a set of hyperparameters. The population is ranked into a social hierarchy: the alpha ($\alpha$) is the best solution, followed by the beta ($\beta$) and delta ($\delta$) as the second and third best solutions, respectively. All other solutions are designated as omega ($\omega$). The optimization process is guided by the three leading wolves, which direct the search toward promising regions of the hyperparameter space.

The hunting behavior is mathematically modeled by updating the position of each candidate solution based on the positions of the three leaders. The distance vectors from the current wolf to the alpha, beta, and delta wolves are first calculated:

3 $$\begin{aligned} D_\alpha = |C_1 \cdot X_\alpha - X_i|, \quad D_\beta = |C_2 \cdot X_\beta - X_i|, \quad D_\delta = |C_3 \cdot X_\delta - X_i| \end{aligned}$$

Each candidate solution’s new position, $X_i(t+1)$, is then determined by averaging the positions of the three leaders, adjusted by these distance vectors:

4 $$\begin{aligned} X_{i}(t+1) = \frac{(X_\alpha - A_1 \cdot D_\alpha ) + (X_\beta - A_2 \cdot D_\beta ) + (X_\delta - A_3 \cdot D_\delta )}{3} \end{aligned}$$

The coefficient vectors *A* and *C* introduce stochasticity and control the balance between exploration and exploitation. They are defined as $A = 2a \cdot r_1 - a$ and $C = 2 \cdot r_2$, where $r_1$ and $r_2$ are random vectors sampled uniformly from [0, 1], and *a* is a control parameter that decreases linearly from 2 to 0 over the course of iterations. Here, $X_i$ represents the current position of a candidate solution in the hyperparameter search space, while $X_\alpha$, $X_\beta$, and $X_\delta$ denote the positions of the three leading solutions.

**Algorithm 2:**
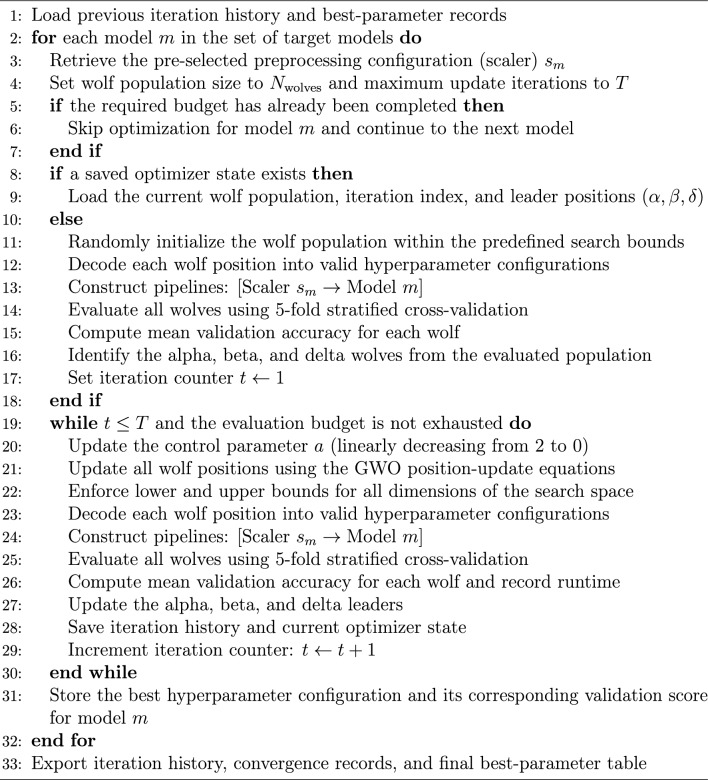
Experiment-specific GWO optimization procedure

In this study, GWO was applied within the same experimental pipeline as TPE, using model-specific preprocessing and stratified cross-validation. Similar to TPE, the optimization process supported checkpoint-based resumption to improve computational efficiency. Each candidate hyperparameter configuration was evaluated using 5-fold stratified cross-validation on the training set, and the fitness of each wolf was defined as the mean validation accuracy across the five folds. Algorithm 2 summarizes the experiment-specific optimization procedure adopted in this study.

#### Hyperparameter search space

Each of the 14 models was optimized using both TPE and GWO under a unified experimental setting, with performance assessed using 5-fold stratified cross-validation on the training set. For TPE, the optimization budget was defined as 50 trials per model. For GWO, the optimization budget was defined in terms of total objective-function evaluations, resulting in 50 evaluations per model, corresponding to the evaluation of the initial wolf population followed by iterative re-evaluation of the updated population.

The search space for each model was defined based on commonly used hyperparameter ranges reported in the literature and practical guidelines for each algorithm family. Continuous, discrete, and categorical parameters were included as appropriate, allowing both optimization methods to explore a heterogeneous and model-specific configuration space.

Table 4 summarizes the hyperparameter search ranges considered for each model.

### Evaluation metrics

Multiple evaluation metrics are used to evaluate model performance, each revealing different aspects of classification behavior. Accuracy alone is insufficient for imbalanced datasets^51^. Therefore, additional metrics such as precision, recall, F1-score, and Matthews correlation coefficient (MCC) are used to provide a complete understanding of model performance ^52^.

Precision reflects the proportion of predicted positives that are correct, while recall measures the model’s ability to identify all true positive instances. The F1-score balances both. For grid stability, precision is critical since false alarms trigger unnecessary protective actions and operational costs^53^. MCC is particularly suitable for imbalanced datasets, as it incorporates all elements of the confusion matrix and provides a balanced evaluation even when class distributions are uneven.

The area under the receiver operating characteristic curve (AUC) quantifies the model’s ability to discriminate between stable and unstable grid states across different decision thresholds. Log loss and Cohen’s kappa are also reported to assess probabilistic calibration and inter-rater agreement, respectively. When considered together, these metrics provide a comprehensive evaluation framework, enabling a more robust and reliable assessment of model performance.

The mathematical definitions of the evaluation metrics are provided below.

5 $$\begin{aligned} & \text {Accuracy} = \frac{TP + TN}{TP + TN + FP + FN} \end{aligned}$$

6 $$\begin{aligned} & \text {Precision} = \frac{TP}{TP + FP} \end{aligned}$$

7 $$\begin{aligned} & \text {Recall} = \frac{TP}{TP + FN} \end{aligned}$$

8 $$\begin{aligned} & \text {F1-Score} = 2 \times \frac{\text {Precision} \times \text {Recall}}{\text {Precision} + \text {Recall}} \end{aligned}$$

9 $$\begin{aligned} & \text {MCC} = \frac{TP \cdot TN - FP \cdot FN}{\sqrt{(TP + FP)(TP + FN)(TN + FP)(TN + FN)}} \end{aligned}$$

10 $$\begin{aligned} & TPR = \frac{TP}{TP + FN}, FPR = \frac{FP}{FP + TN} \end{aligned}$$

11 $$\begin{aligned} & \text {LogLoss} = -\frac{1}{N} \sum _{i=1}^{N} \left[ y_i \log (p_i) + (1 - y_i)\log (1 - p_i) \right] \end{aligned}$$

12 $$\begin{aligned} & \kappa = \frac{P_o - P_e}{1 - P_e} \end{aligned}$$

$$\begin{aligned} P_o = \frac{TP + TN}{TP + TN + FP + FN}, \quad P_e = \frac{(TP + FP)(TP + FN) + (TN + FN)(TN + FP)}{(TP + TN + FP + FN)^2} \end{aligned}$$

where *TP*, *TN*, *FP*, and *FN* denote the number of true positives, true negatives, false positives, and false negatives, respectively.

$y_i \in \{0,1\}$ represents the true label of the *i*-th sample, $p_i \in [0,1]$ denotes the predicted probability of the positive class, and *N* is the total number of samples.

$P_o$ denotes the observed agreement between predicted and true labels, while $P_e$ represents the expected agreement by chance.

**Table 4:** Hyperparameter search space for each model.

Model	Hyperparameter	Search range
AdaBoost	n_estimators	[50, 500]
	learning_rate	[0.01, 2.0]
	Algorithm	{SAMME, SAMME.R}
CatBoost	Depth	[4, 10]
	learning_rate	[0.01, 0.5]
	Iterations	[100, 500]
	l2_leaf_reg	[1, 10]
	bagging_temperature	[0, 10]
GradientBoosting	n_estimators	[100, 500]
	learning_rate	[0.01, 0.5]
	max_depth	[3, 10]
	Subsample	[0.5, 1.0]
KNN	n_neighbors	[3, 30]
	Weights	{uniform, distance}
	Metric	{euclidean, manhattan}
LDA	Solver	{svd, lsqr, eigen}
LightGBM	num_leaves	[20, 100]
	max_depth	[5, 15]
	learning_rate	[0.01, 0.5]
	n_estimators	[100, 500]
	Subsample	[0.5, 1.0]
	colsample_bytree	[0.5, 1.0]
LinearSVC	C	[0.01, 100]
	Loss	{hinge, squared_hinge}
LogisticRegression	C	[0.01, 10]
	Penalty	{l1, l2}
NaiveBayes	var_smoothing	$[10^{-12}, 10^{-6}]$
QDA	reg_param	[0, 1]
RandomForest	n_estimators	[100, 500]
	max_depth	[5, 50]
	min_samples_split	[2, 10]
SGD	Alpha	$[10^{-6}, 10^{-2}]$
	l1_ratio	[0, 1]
	Penalty	{l1, l2, elasticnet}
SVC	C	[0.1, 1000]
	Kernel	{rbf, poly}
	Gamma	{scale, auto}
XGBoost	max_depth	[3, 10]
	learning_rate	[0.01, 0.3]
	n_estimators	[100,500]
	Subsample	[0.5, 1.0]
	colsample_bytree	[0.5, 1.0]

### Explainable AI (XAI) methods

We employ SHAP to interpret model predictions within our safety-critical application, utilizing its rigorous game-theoretic foundation to quantify feature contributions^54^

Initially, the analysis focuses on global importance through the *SHAP summary plot*. which ranks features by their average impact on model outputs to identify which variables drive predictions most consistently across the dataset^55^. This visualization clarifies the direction of impact, specifically whether features push the model toward stability or instability. Building upon this global view, we utilize *SHAP Dependence Plot* to reveal how individual feature values relate to their specific contributions. These plots are essential for identifying non-monotonic or U-shaped relationships, thereby detecting critical thresholds where feature behavior changes significantly^56^.

In addition to these global insights, we examine individual instances using the *SHAP Waterfall Plot* to trace how specific features drive a single prediction^57^. This approach is grounded in the mathematical decomposition:

13 $$\begin{aligned} f(x) = E[f(x)] + \sum _{i=1}^{n} \phi _i(x) \end{aligned}$$

where *f*(*x*) represents the model output, *E*[*f*(*x*)] is the baseline average prediction and $\phi _i(x)$ is the SHAP contribution of feature *i*. By applying this formula, we can precisely attribute the model’s final output to the *n* constituent features.

To further validate these patterns, we incorporate *PDP* and *ICE* curves to observe how feature effects vary across different operating conditions^41^. While PDPs illustrate the average effect of a feature across all instances, ICE plots provide a more detailed view by showing how predictions change for individual cases. Specifically, the degree of parallelism among ICE lines indicates whether feature effects are purely additive or influenced by complex interactions.

Finally, by synthesizing these four methodologies, we can examine whether the model’s decisions are consistent with relationships suggested by power system theory. Thus, this multi-layered interpretation indicates that the framework captures structured and stable patterns in the data rather than relying on spurious correlations^58^.

## Results and discussion

This section shows the results of ML models on the SG prediction task and the results of data preprocessing.

### Exploratory data analysis and feature selection results

**Fig. 3:**
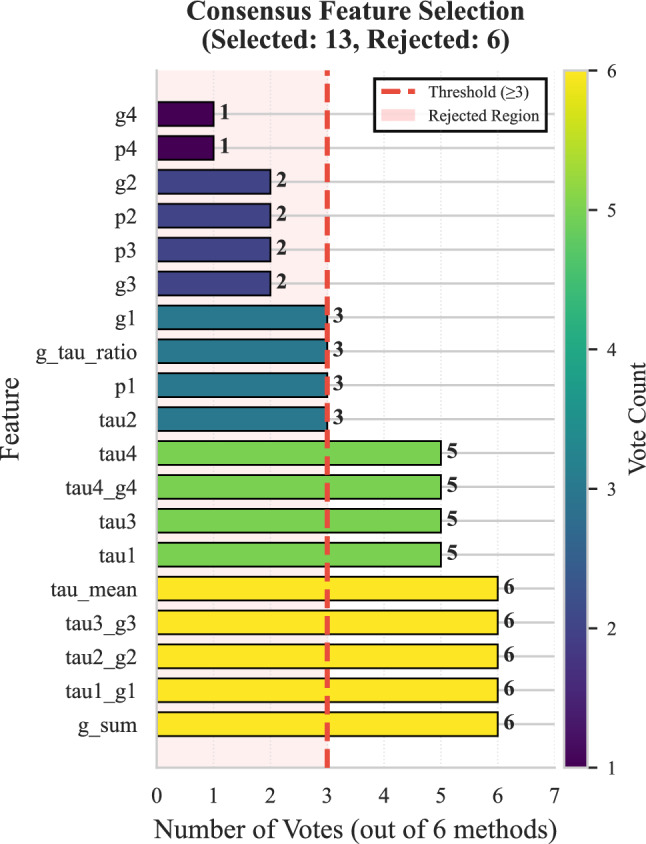
Feature selection results.

Applying the consensus feature selection procedure retained 13 of the 19 candidate features (31.6% reduction). Figure 3 shows the voting distribution across the six selection methods. Five features received unanimous support from all methods, four received five votes, and four met the minimum consensus threshold of three votes.

**Fig. 4:**
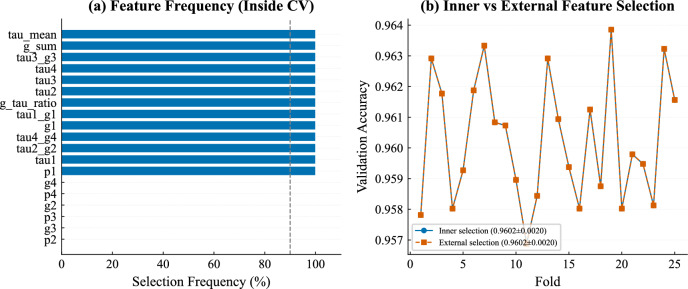
(**a**) Feature selection frequency across 5$\times$5 repeated cross-validation folds. (**b**) Validation accuracy comparison between inner-fold and externally selected features.

To evaluate the stability of the selected feature subset, the feature selection procedure was repeated independently within each fold of a repeated stratified cross-validation (5$\times$5 folds). As shown in Fig. 4a, the same set of 13 features was consistently selected in all folds, yielding a mean Jaccard similarity of 1.00 with zero variance.

Furthermore, Fig. 4b compares validation accuracy obtained using fold-specific feature selection and the externally selected feature set. The two approaches yielded the same validation performance (96.02% ± 0.20% in both cases), with an absolute difference of 0.00 percentage points. This confirms that the selected features are highly stable and that performing feature selection once on the training data does not introduce measurable bias.

The retained set includes both original variables and engineered interaction features. In particular, the interaction terms ($\tau _1 g_1$, $\tau _2 g_2$, $\tau _3 g_3$, $\tau _4 g_4$) and the aggregated descriptors ($\tau _{\text {mean}}$, $g_{\text {sum}}$) consistently received the highest number of votes. This indicates that relationships between response delays and elasticity parameters provide useful information for distinguishing stable and unstable grid states.

Conversely, several individual consumer parameters (e.g., $p_2$, $p_3$, $p_4$, $g_2$, $g_3$, $g_4$) were rejected by most methods. This suggests that isolated parameters carry less discriminative information than variables that summarize or combine multiple aspects of the system’s behavior.

**Fig. 5:**
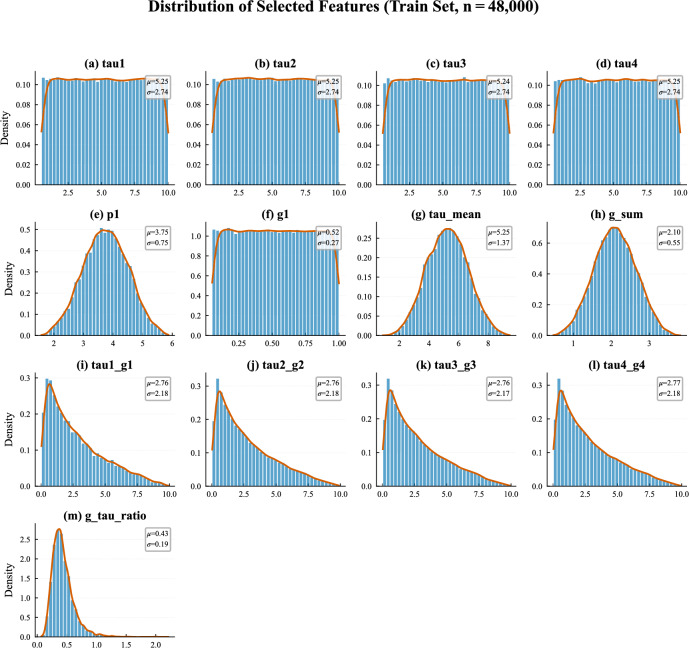
Histograms of the 13 selected features.

The thirteen selected features show markedly different distributions in the training data (Fig. 5), reflecting the mix of original and engineered features. The reaction time parameters (tau1–tau4) and producer elasticity (g1) are roughly uniformly distributed, consistent with the simulation setup. In contrast, the engineered aggregates (tau_mean, g_sum) are approximately normally distributed. The coupling terms and ratio-based features are right-skewed. This heterogeneity motivates the preliminary scaling step described in the methodology.

**Fig. 6:**
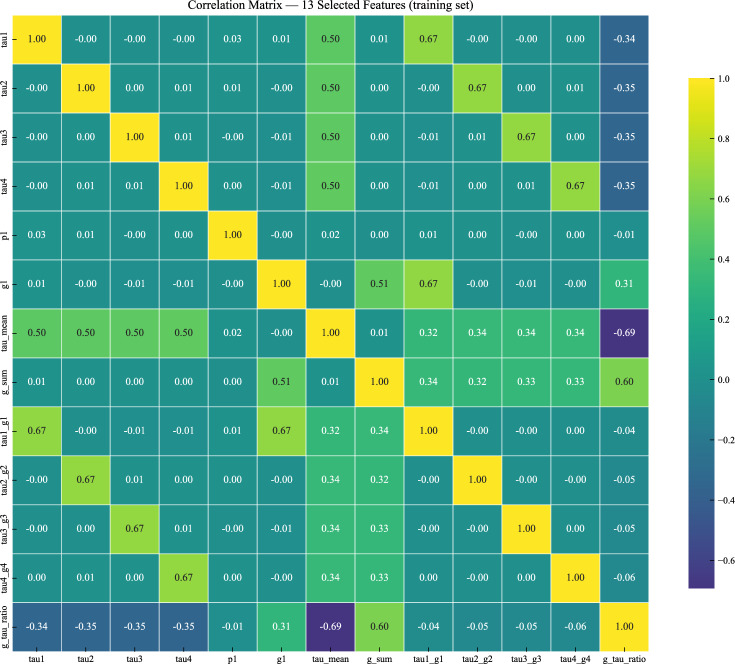
Correlation matrix of the 13 selected features.

The correlation matrix (Fig. 6) shows that the chosen features remain reasonably independent, with a maximum absolute pairwise correlation of 0.67. This lack of strong correlations supports model interpretability. The moderate correlations that do appear (e.g., tau_mean vs. individual tau) are expected due to feature construction. These structured dependencies reflect transparent mathematical relationships rather than problematic redundancy.

**Figure 7:**
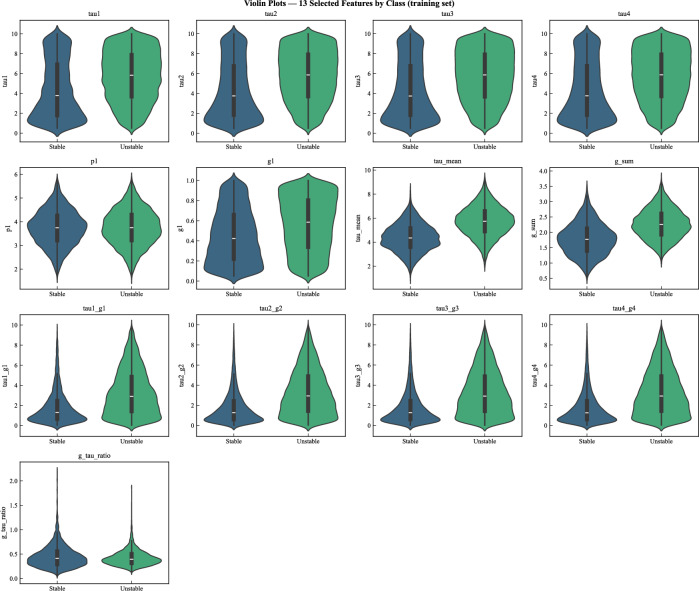
Violin plots of the 13 selected features.

Figure 7 shows distributional differences between stable and unstable grid states. Unstable states tend to concentrate at higher values for reaction times (tau), aligning with power systems theory. Engineered coupling terms exhibit similar separation patterns, where higher values correspond to stronger interactions between response delay and elasticity parameters. This visual evidence demonstrates that the selected feature set contains substantial discriminative information.

Furthermore, the overlapping distributions in the violin plots (Fig. 7) suggest that linear models will likely struggle to find a clear decision boundary, hinting at the need for more complex, non-linear models to capture the underlying patterns. This provides a strong rationale for including tree-based ensembles and other non-linear classifiers in our benchmark.

The training set comprises 30,624 stable instances (63.8%) and 17,376 unstable instances (36.2%), reflecting the moderate class imbalance present in the dataset. This level of imbalance is moderate and does not necessarily require mandatory rebalancing, but its potential impact on model training was nevertheless evaluated. To examine whether this imbalance could influence model training, we conducted preliminary experiments using the synthetic minority oversampling technique (SMOTE). The resampling procedure was applied only to the training data in order to avoid information leakage, and model performance was compared with results obtained using the original class distribution.

**Fig. 8:**
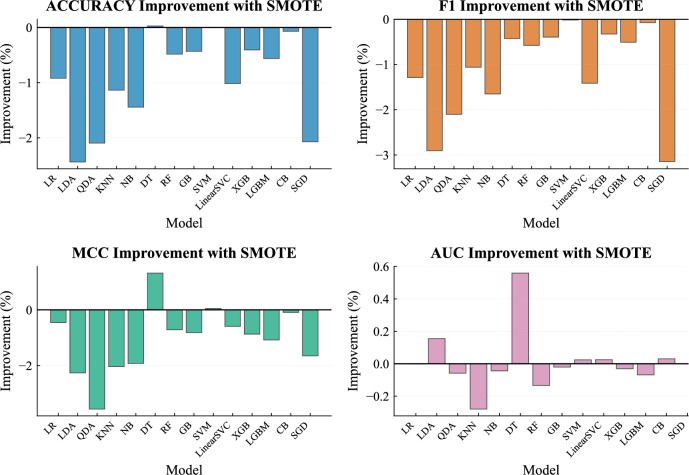
Performance change after applying SMOTE across the evaluated models.

Figure 8 shows the relative performance change across the evaluated models for four metrics: accuracy, F1-score, MCC, and AUC. Positive values indicate performance improvement after resampling, whereas negative values indicate performance degradation. Overall, SMOTE did not consistently improve performance across the evaluated models, with an average performance change of approximately -0.79 percentage points. Consequently, the remaining experiments were conducted using the original dataset without additional balancing.

This imbalance also confirms that accuracy alone is an insufficient metric, which justifies our use of AUC, F1-score, and MCC in all subsequent evaluations.

### Feature space separability analysis

To further assess the impact of feature engineering and feature selection, we examined how the data representation varies across three spaces: the original 12-feature space, the expanded 19-feature engineered space, and the final 13-feature selected space. The goal of this analysis is to confirm that the proposed features enhance the encoding of stability-related structure without artificially simplifying the underlying classification problem.

**Fig. 9:**
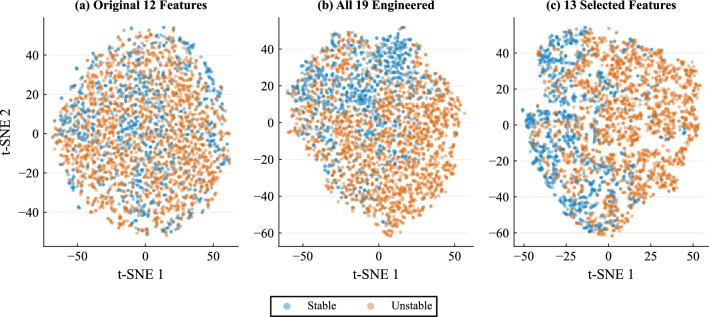
t-SNE projections of the dataset in the original, engineered, and selected feature spaces.

Figure 9 presents t-SNE projections of the three feature spaces. In the original 12-feature representation, stable and unstable samples are highly intermixed, indicating that the classes are not trivially separable. After feature engineering, a clearer structure begins to emerge, and this structure becomes more pronounced after feature selection. Nevertheless, substantial overlap remains in all three projections, showing that the task still requires non-linear decision boundaries.

To complement this visual analysis, we evaluated a linear logistic regression classifier on the three feature representations. The results are reported in Table 5. Using the original 12 features, logistic regression achieved a test accuracy of 81.95%. This increased to 85.98% when all 19 engineered features were used, and to 86.04% after feature selection. The improvement confirms that the engineered variables add useful information to the representation. At the same time, the overall performance of the linear classifier remains well below that of the best non-linear models, indicating that the problem is still not linearly separable.

**Table 5:** Logistic regression performance across feature spaces.

Feature set	Train accuracy	Test accuracy
12 original features	0.8133	0.8195
19 engineered features	0.8566	0.8598
13 selected features	0.8565	0.8604

**Fig. 10:**
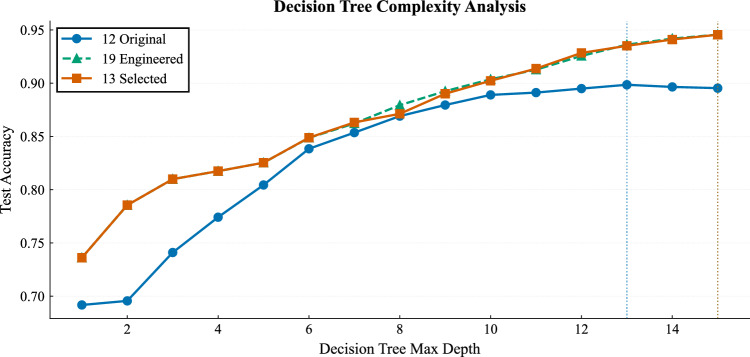
Decision tree test accuracy as a function of maximum depth across the three feature spaces.

We also assessed the complexity of the decision boundary using a decision tree classifier with varying maximum depth. Figure 10 shows the relationship between tree depth and test accuracy for the three feature spaces. For the original 12 features, the best performance was obtained at a depth of 13 with a test accuracy of 89.85%. For both the 19 engineered features and the 13 selected features, the optimal depth increased to 15, with test accuracies of 94.56% and 94.55%, respectively. If the engineered variables had made the problem trivial, a shallow tree would have been sufficient. Instead, the requirement for deep trees indicates that high performance still depends on complex, hierarchical, non-linear feature interactions.

Taken together, these results show that feature engineering and feature selection improve the organization of the feature space and make stability-related structure more accessible to learning algorithms. However, they do not collapse the task into trivial separability. The classification problem remains fundamentally non-linear and still requires complex decision boundaries.

### Feature scaling results

**Fig. 11:**
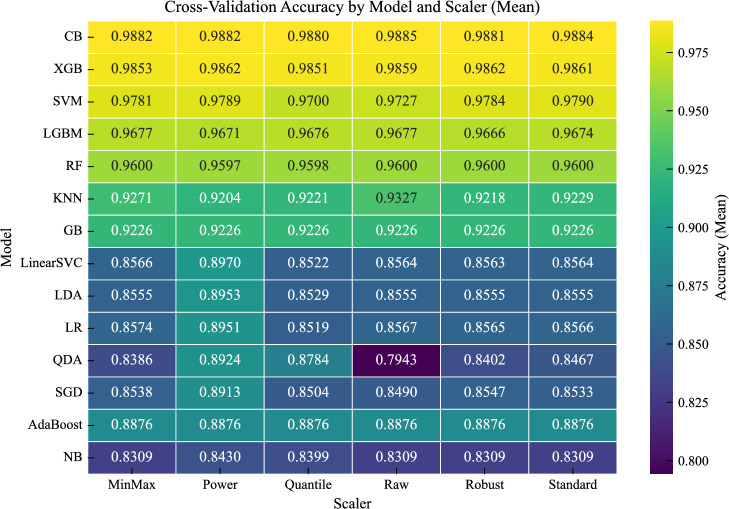
Mean cross-validation accuracy for all model-scaler combinations.

**Fig. 12:**
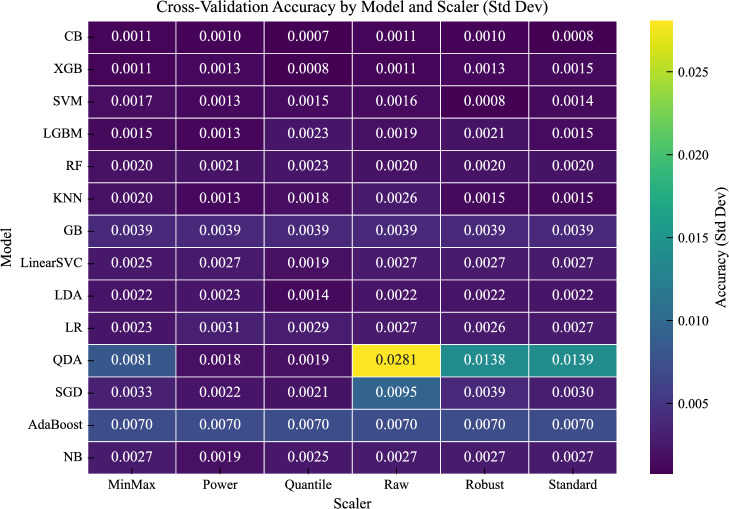
Standard deviation of cross-validation accuracy across folds for all model-scaler combinations.

**Fig. 13:**
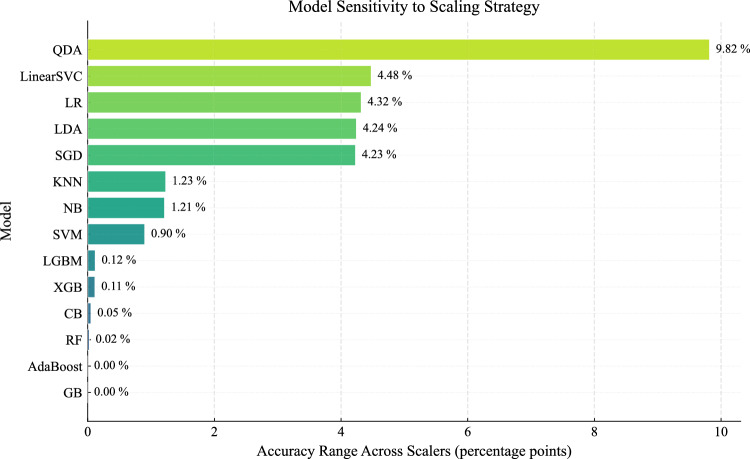
Sensitivity of each model to feature scaling, measured as the variance of performance across scaling strategies.

The optimal scaling strategies, determined using 5-fold stratified cross-validation on the training set, are summarized in Table 6. Figure 11 presents the mean cross-validation accuracy for all model–scaler combinations, while Fig. 12 shows the corresponding variability across folds. Figure 13 further summarizes the sensitivity of each model to feature scaling, measured as the variance of performance across scaling strategies.

The results show that the effectiveness of feature scaling depends strongly on model architecture. Tree-based ensemble methods (e.g., CatBoost, RandomForest, GradientBoosting) exhibit minimal variation across scaling strategies, as observed in both the mean and standard deviation heatmaps. Their performance remains stable regardless of preprocessing, reflecting their inherent invariance to feature scale due to split-based decision mechanisms. In some cases, such as LightGBM, multiple scaling strategies (including raw and transformed features) yield very similar performance, with differences falling within the variability observed across cross-validation folds. This indicates that scaling has a negligible effect on these models.

In contrast, linear and probabilistic models (e.g., Logistic Regression, LDA, QDA, SGD) are clearly sensitive to preprocessing. For these models, the PowerTransformer consistently provides the best performance. This can be attributed to variance stabilization and reduced skewness, which better align feature distributions with model assumptions and improve optimization stability^42^. This sensitivity is also reflected in Fig. 13, where these models show the largest variation across scaling strategies.

Kernel-based methods (SVC) show moderate sensitivity, with StandardScaler yielding the best results. This behavior is consistent with the reliance of RBF kernels on distance computations in a normalized feature space.

Interestingly, the distance-based KNN model performs best on the raw features, suggesting that the original feature scales already preserve meaningful relationships in the input space and that additional transformations may distort the distance structure.

The standard deviation heatmap (Fig. 12) supports these observations: tree-based models exhibit consistently low variability across folds, whereas linear and probabilistic models show higher variability. Despite this sensitivity, appropriate scaling (particularly PowerTransformer) leads to both improved and stable performance.

Overall, these results confirm that scaling effects are model-dependent and that a single global preprocessing strategy is suboptimal. Selecting scaling strategies tailored to model characteristics leads to more reliable and consistent performance, in line with recent empirical findings^42^.

**Table 6:** Optimal feature scaling strategy for each model based on 5-fold cross-validation. Values reported as Mean Accuracy ± Std (%).

Model	Optimal scaler	Mean accuracy ± Std (%)
LR	Power	89.53 ± 0.31
LDA	Power	89.53 ± 0.23
QDA	Power	89.24 ± 0.18
NB	Power	84.30 ± 0.19
KNN	Raw	93.27 ± 0.26
LinearSVC	Power	89.70 ± 0.27
SVM	Standard	97.90 ± 0.14
AdaBoost	Raw	88.76 ± 0.70
RF	MinMax	96.00 ± 0.20
GB	Raw	92.26 ± 0.39
XGB	MinMax	96.90 ± 0.28
LGBM	Raw	96.78 ± 0.19
CB	MinMax	93.93 ± 0.17
SGD	Power	89.13 ± 0.22

### Hyperparameter optimization results

The performance of all models under TPE and GWO optimization is presented in Tables 7 and 8. Results are reported for both training and test sets using accuracy, MCC, and AUC, together with computational cost in terms of optimization time and evaluation time.

Across both tables, a consistent pattern emerges. Tree-based ensemble models (LGBM, GB, CB, and XGB) achieve the highest predictive performance, with near-identical results across all three metrics. In particular, LGBM attains the best overall performance, reaching the highest accuracy, MCC, and AUC on the test set.

While accuracy values are consistently high across top-performing models, MCC provides a more reliable assessment of classification quality. The high MCC values observed for ensemble models confirm that their performance reflects balanced and robust predictions rather than dominance of a single class.

At the same time, the results show that several models achieve very similar performance levels, with overlapping confidence intervals and only marginal differences between them. This indicates that performance is approaching saturation under the current feature representation, and that different model–optimizer combinations converge to similarly effective solutions.

The observed high performance should be interpreted in the context of the underlying feature representation. As shown in the "Feature space separability analysis" Section, the feature space still exhibits overlap between classes and is not linearly separable. In this setting, the strong performance of ensemble models is consistent with their ability to capture complex non-linear relationships, rather than indicating trivial class separability.

From a computational perspective, substantial differences are observed between models. More complex models, such as SVM and GB, require significantly higher optimization time, whereas simpler models (e.g., LR, LDA, NB) exhibit much lower computational cost but reduced predictive performance. Evaluation time follows a similar trend, with ensemble models maintaining relatively low inference cost despite their higher optimization overhead.

Comparing TPE and GWO, both strategies produce highly similar results across all performance metrics. However, GWO generally exhibits lower optimization time for several models, indicating more efficient convergence. This difference suggests that, once performance saturation is reached, the choice of optimization strategy becomes more relevant from a computational perspective than from a predictive one.

Overall, the results in Tables 7 and 8 indicate that model performance is largely stabilized, and that the primary distinction between TPE and GWO lies in computational efficiency rather than predictive accuracy.

**Table 7:** Performance of TPE-optimized models. Values: mean % (95% CI).

Model	Split	Accuracy (%)	MCC (%)	AUC (%)	HPO time (s)	Eval. time (s)
LR	Train	89.73 (89.46–90.01)	77.68 (77.10–78.29)	96.60 (96.47–96.73)	495.86	136.34
	Test	90.56 (90.04–91.08)	79.45 (78.30–80.62)	96.86 (96.61–97.11)		
LDA	Train	89.53 (89.25–89.80)	77.26 (76.67–77.85)	96.50 (96.37–96.63)	252.95	123.84
	Test	90.35 (89.83–90.89)	78.99 (77.86–80.16)	96.75 (96.50–97.00)		
QDA	Train	89.90 (89.62–90.18)	77.90 (77.31–78.50)	96.71 (96.58–96.84)	237.93	110.39
	Test	90.59 (90.08–91.09)	79.45 (78.35–80.57)	96.97 (96.73–97.22)		
NB	Train	84.30 (83.98–84.65)	66.34 (65.64–67.07)	92.37 (92.15–92.61)	184.29	90.95
	Test	84.75 (84.12–85.39)	67.25 (65.89–68.62)	92.74 (92.30–93.17)		
KNN	Train	94.88 (94.67–95.08)	88.88 (88.44–89.31)	98.93 (98.87–99.00)	1720.24	302.90
	Test	95.42 (95.04–95.78)	90.04 (89.22–90.85)	99.14 (99.01–99.24)		
LSVC	Train	89.73 (89.46–90.01)	77.71 (77.13–78.32)	96.60 (96.47–96.73)	726.57	203.28
	Test	90.49 (89.96–91.03)	79.32 (78.19–80.48)	96.85 (96.60–97.10)		
SVM	Train	99.44 (99.36–99.50)	98.78 (98.62–98.92)	99.97 (99.96–99.98)	28416.02	1322.29
	Test	99.62 (99.51–99.73)	99.19 (98.94–99.42)	99.98 (99.96–99.99)		
AdaB	Train	95.10 (94.91–95.28)	89.39 (88.99–89.79)	99.13 (99.08–99.18)	8083.07	471.18
	Test	95.07 (94.68–95.46)	89.31 (88.49–90.16)	99.16 (99.06–99.26)		
RF	Train	97.06 (96.91–97.21)	93.62 (93.29–93.94)	99.62 (99.58–99.65)	5693.33	394.22
	Test	97.92 (97.68–98.17)	95.50 (94.98–96.03)	99.79 (99.74–99.83)		
GB	Train	99.58 (99.52–99.64)	99.09 (98.97–99.21)	99.98 (99.98–99.99)	44215.38	793.89
	Test	99.80 (99.72–99.88)	99.57 (99.39–99.73)	99.99 (99.99–100.00)		
XGB	Train	99.36 (99.28–99.42)	98.61 (98.44–98.75)	99.97 (99.96–99.98)	802.20	139.34
	Test	99.54 (99.42–99.66)	99.01 (98.74–99.26)	99.99 (99.98–99.99)		
LGBM	Train	99.77 (99.73–99.81)	99.50 (99.41–99.59)	99.99 (99.99–100.00)	2784.90	212.04
	Test	**99.95** (99.91–99.98)	**99.89** (99.80–99.96)	**100** (100–100)		
CB	Train	99.40 (99.33–99.47)	98.71 (98.55–98.85)	99.97 (99.96–99.98)	2291.14	142.13
	Test	99.62 (99.50–99.72)	99.17 (98.92–99.39)	99.99 (99.98–100)		
SGD	Train	89.61 (89.33–89.89)	77.41 (76.82–78.03)	96.51 (96.38–96.65)	265.62	103.45
	Test	90.28 (89.75–90.82)	78.77 (77.61–79.94)	96.83 (96.57–97.08)

**Table 8:** Performance of GWO-optimized models. Values: mean % (95% CI).

Model	Split	Accuracy (%)	MCC (%)	AUC (%)	HPO time (s)	Eval. time (s)
LR	Train	89.73 (89.46–90.01)	77.68 (77.09–78.29)	96.60 (96.47–96.73)	511.65	132.91
	Test	90.56 (90.04–91.08)	79.45 (78.30–80.62)	96.86 (96.61–97.11)		
LDA	Train	89.53 (89.25–89.80)	77.26 (76.67–77.85)	96.50 (96.37–96.63)	329.58	152.68
	Test	90.35 (89.83–90.89)	78.99 (77.86–80.16)	96.75 (96.50–97.00)		
QDA	Train	89.86 (89.59–90.15)	77.83 (77.23–78.41)	96.74 (96.62–96.87)	286.29	122.48
	Test	90.52 (90.01–91.04)	79.28 (78.16–80.42)	97.01 (96.76–97.25)		
NB	Train	84.30 (83.98–84.65)	66.34 (65.64–67.07)	92.37 (92.15–92.61)	285.88	138.37
	Test	84.75 (84.12–85.39)	67.25 (65.89–68.62)	92.74 (92.30–93.17)		
KNN	Train	94.82 (94.61–95.02)	88.75 (88.29–89.19)	98.94 (98.88–99.00)	1709.73	148.48
	Test	95.43 (95.06–95.81)	90.08 (89.28–90.90)	99.16 (99.05–99.27)		
LSVC	Train	89.71 (89.45–89.99)	77.66 (77.08–78.28)	96.59 (96.47–96.73)	360.86	105.38
	Test	90.57 (90.04–91.09)	79.48 (78.37–80.64)	96.85 (96.60–97.10)		
SVM	Train	99.45 (99.38–99.51)	98.80 (98.65–98.93)	99.97 (99.96–99.98)	25435.12	1219.47
	Test	99.71 (99.61–99.80)	99.37 (99.15–99.57)	99.98 (99.96–99.99)		
AdaB	Train	94.08 (93.87–94.28)	87.14 (86.69–87.59)	98.77 (98.70–98.83)	3574.22	423.28
	Test	93.99 (93.56–94.41)	86.94 (85.98–87.84)	98.84 (98.71–98.96)		
RF	Train	97.33 (97.18–97.47)	94.20 (93.87–94.51)	99.66 (99.63–99.69)	11977.41	972.64
	Test	98.01 (97.77–98.25)	95.68 (95.14–96.21)	99.82 (99.78–99.86)		
GB	Train	99.67 (99.61–99.71)	99.28 (99.17–99.38)	99.99 (99.98–99.99)	40764.22	731.89
	Test	99.78 (99.69–99.86)	99.51 (99.33–99.69)	100 (100–100)		
XGB	Train	99.36 (99.29–99.43)	98.62 (98.47–98.77)	99.97 (99.97–99.98)	434.49	92.11
	Test	99.73 (99.64–99.83)	99.42 (99.22–99.62)	99.98 (99.97–99.99)		
LGBM	Train	99.74 (99.70–99.79)	99.44 (99.34–99.53)	99.99 (99.99–100)	1207.98	138.05
	Test	**99.90** (99.84–99.95)	**99.78** (99.66–99.89)	**100** (100–100)		
CB	Train	99.44 (99.37–99.51)	98.80 (98.64–98.93)	99.98 (99.97–99.98)	2653.54	161.80
	Test	99.69 (99.58–99.78)	99.33 (99.10–99.53)	99.99 (99.99–100)		
SGD	Train	89.55 (89.28–89.83)	77.29 (76.70–77.88)	96.33 (96.18–96.47)	241.71	97.61
	Test	90.13 (89.61–90.67)	78.48 (77.35–79.66)	96.62 (96.34–96.89)		

#### Statistical significance analysis

To assess whether the observed performance differences are statistically meaningful, paired comparisons were conducted on the set of models for which results were available under both optimization strategies.

As summarized in Table 9, the Wilcoxon signed-rank test indicates that the difference between TPE and GWO is not statistically significant ($p = 0.8438$). A paired t-test leads to the same conclusion ($p = 0.3977$).

In contrast, both optimized configurations significantly outperform the baseline, with p-values below 0.01. These results confirm that while HPO provides substantial improvements over baseline models, the choice between TPE and GWO does not lead to statistically significant differences in predictive performance.

**Table 9:** Statistical comparison between optimization strategies using representative models.

Comparison	Test	p-value
TPE vs GWO	Wilcoxon	0.8438
TPE vs GWO	Paired t-test	0.3977
Baseline vs TPE	Wilcoxon	0.0029
Baseline vs GWO	Wilcoxon	0.0039

### Ablation study

To better understand the contribution of each stage in the proposed SG stability pipeline, an ablation study was conducted using the LightGBM model under seven configurations. These configurations vary both the feature representation and the optimization strategy, enabling a structured comparison of how feature construction, feature reduction, and hyperparameter tuning affect predictive performance on the held-out test set.

The evaluated configurations are summarized in Table 10. Each configuration represents a specific combination of feature representation and optimization strategy, allowing a consistent comparison across all experimental settings.

**Table 10:** Description of ablation configurations used in the study.

ID	Configuration description
A	Raw features (12) with default parameters
B	Engineered features (19) with default parameters
F	Engineered features (19) with TPE optimization
G	Engineered features (19) with GWO optimization
C	Selected features (13) with baseline settings
D	Selected features (13) with TPE optimization
E	Selected features (13) with GWO optimization

The numerical results for all configurations are presented in Table 11. Moving from the raw baseline to the engineered feature space leads to a clear improvement in both accuracy and MCC, indicating that the constructed variables better capture relationships associated with grid stability. Applying HPO to the engineered features results in a substantial additional gain, with both optimization strategies reaching near-perfect performance across all evaluation metrics.

When considering the reduced feature subset, the baseline configuration improves over the raw features but remains below the optimized engineered models. Once optimization is applied, configuration D achieves the best overall performance on the test set, while configuration E remains very close. This suggests that performance is largely determined by the quality of the feature representation, with only minor differences between optimization strategies at this stage.

**Table 11:** Ablation results for LightGBM configurations. Metrics (except LL) are (%). 95% CI shown below estimates.

ID	Acc (CI)	Prec (CI)	Rec (CI)	F1 (CI)	AUC (CI)	LL (CI)	Kappa (CI)	MCC (CI)
A	96.03 (95.6–96.3)	95.93 (95.5–96.3)	97.92 (97.5–98.2)	96.92 (96.6–97.1)	99.46 (99.3–99.5)	0.1290 (.12–.13)	91.33 (90.5–92.0)	91.36 (90.6–92.1)
B	97.43 (97.1–97.7)	97.49 (97.1–97.8)	98.51 (98.2–98.7)	98.00 (97.7–98.2)	99.72 (99.6–99.7)	0.1017 (.09–.10)	94.42 (93.7–95.0)	94.43 (93.7–95.0)
F	99.88 (99.8–99.9)	99.86 (99.7–99.9)	99.95 (99.8–99.9)	99.90 (99.8–99.9)	100.0 (100–100)	0.0038 (.00–.00)	99.73 (99.5–99.8)	99.73 (99.5–99.8)
G	99.90 (99.8–99.9)	99.88 (99.8–99.9)	99.96 (99.9–100)	99.92 (99.8–99.9)	100.0 (100–100)	0.0023 (.00–.00)	99.78 (99.6–99.8)	99.78 (99.6–99.8)
C	97.11 (96.8–97.4)	97.15 (96.7–97.5)	98.35 (98.0–98.6)	97.75 (97.5–97.9)	99.69 (99.6–99.7)	0.1069 (.10–.11)	93.71 (93.0–94.3)	93.72 (93.0–94.3)
D	**99.95** (99.9–99.9)	**99.95** (99.8–99.9)	**99.97** (99.9–100)	**99.96** (99.9–99.9)	**100.0** (100–100)	**0.0022** (.00–.00)	**99.89** (99.8–99.9)	**99.89** (99.8–99.9)
E	99.90 (99.8–99.9)	99.88 (99.8–99.9)	99.96 (99.9–100)	99.92 (99.8–99.9)	100.0 (100–100)	0.0027 (.00–.00)	99.78 (99.6–99.8)	99.78 (99.6–99.8)

**Fig. 14:**
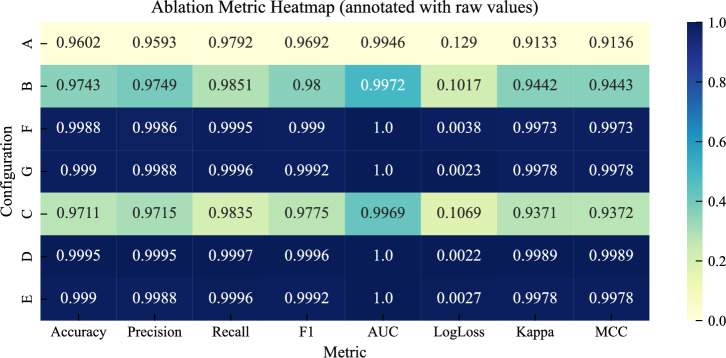
Ablation heatmap for the seven LightGBM configurations across the main evaluation metrics.

**Fig. 15:**
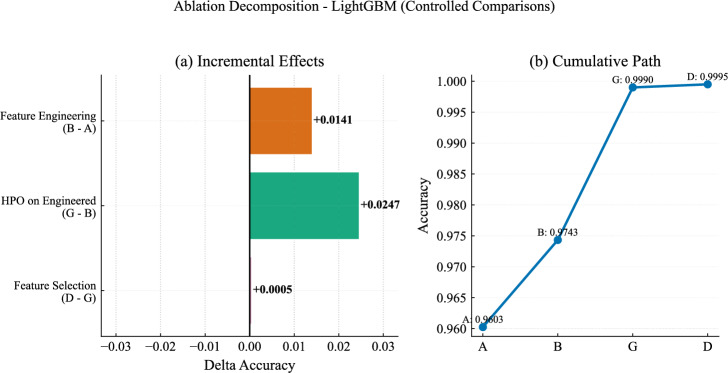
Incremental and cumulative accuracy changes across the pipeline, illustrating the progression from the baseline configuration to the final optimized model.

Figure 14 summarizes the performance across all configurations, showing a consistent improvement across all evaluation metrics. This overall trend is clarified in Fig. 15, which traces the progression from the baseline configuration to the final optimized model. The initial improvement reflects the effect of feature construction, followed by a larger gain from HPO, while the final step contributes only a marginal refinement.

To support these observations, Table 12 reports pairwise accuracy differences based on bootstrap analysis. The results confirm the contribution of feature engineering and the gains achieved through optimization. Among the strongest configurations, the differences become small, with configurations E and G showing nearly identical performance, while configuration D maintains a slight advantage. Although several comparisons are statistically significant, their magnitude is very small, indicating that performance has largely saturated at this stage.

Overall, the ablation results show that the performance gains mainly come from feature engineering and HPO, while feature selection provides a final refinement. While several configurations achieve near-perfect results, configuration D is selected as the final model since it consistently achieves the best performance across all metrics. To ensure that this conclusion is not tied to a specific optimization method, configuration E, which uses the same selected features with GWO tuning, is also included for comparison in the final performance analysis.

**Table 12:** Pairwise accuracy differences between key ablation configurations.

Comparison	Mean $\Delta$ accuracy	95% CI	Significant at 95%
B vs A	+ 0.0141	[0.0108, 0.0175]	Yes
D vs A	+ 0.0392	[0.0358, 0.0427]	Yes
E vs A	+ 0.0387	[0.0353, 0.0422]	Yes
F vs B	+ 0.0244	[0.0217, 0.0273]	Yes
G vs B	+ 0.0246	[0.0218, 0.0276]	Yes
D vs F	+ 0.0007	[0.0002, 0.0013]	Yes
E vs G	− 0.0000	[− 0.0005, 0.0005]	No
D vs E	+ 0.0005	[0.0001, 0.0010]	Yes

### Final model performance

The final performance of the selected model is examined in detail, with configuration D as the primary reference and configuration E included for comparison.

**Table 13:** Final LightGBM performance and efficiency comparison (TPE vs. GWO).

Metric	LightGBM-TPE (D)	LightGBM-GWO (E)
Accuracy	**99.95**	99.90
Precision	**99.95**	99.88
Recall	**99.97**	99.96
F1 Score	**99.96**	99.92
AUC	100.0	100.0
Log Loss	**0.0022**	0.0027
Cohen Kappa	**99.89**	99.78
MCC	**99.89**	99.78
Optimization time (s)	2572.0	**1069.6**
Inference latency (ms)	4.44	**2.92**
Batch throughput (samples/s)	**70622**	53367

Table 13 shows that both models achieve near-identical predictive performance across all evaluation metrics. The TPE-based model provides slightly higher accuracy, MCC, and kappa values, although the differences remain very small.

**Fig. 16:**
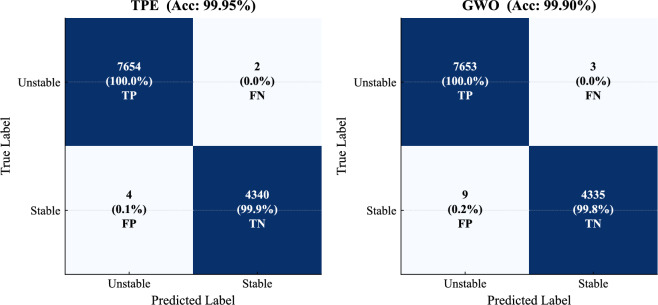
Confusion matrices for the final LightGBM models.

A more operational perspective is obtained from the confusion matrices in Fig. 16. The TPE model produces only 6 misclassifications (2 false negatives and 4 false positives), while the GWO model results in 12 misclassifications (3 false negatives and 9 false positives).

False negatives correspond to unstable conditions incorrectly classified as stable, which may prevent timely corrective actions and increase the risk of cascading failures. False positives represent stable conditions incorrectly classified as unstable, potentially triggering unnecessary control actions. The very low number of such errors indicates strong operational reliability, with the TPE model showing a slight advantage.

**Fig. 17:**
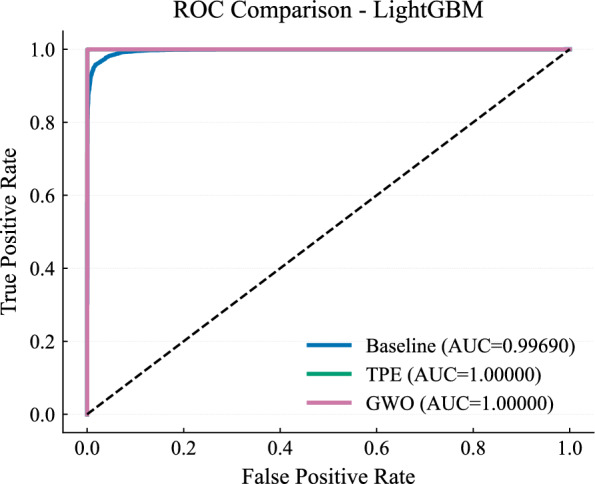
ROC curves for LightGBM models.

Figure 17 confirms that both models achieve near-complete class separability, with overlapping ROC curves and an AUC of 1.000. This allows consistent performance across a wide range of decision thresholds.

**Fig. 18:**
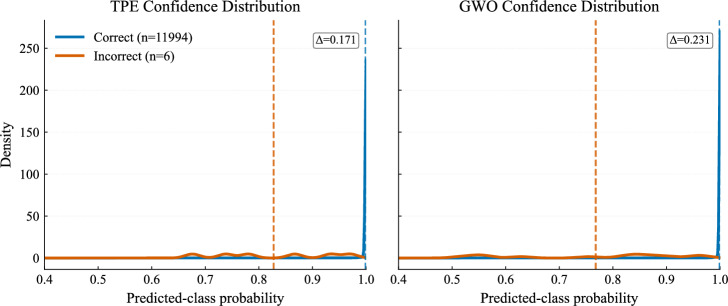
Confidence distribution of predictions.

**Fig. 19:**
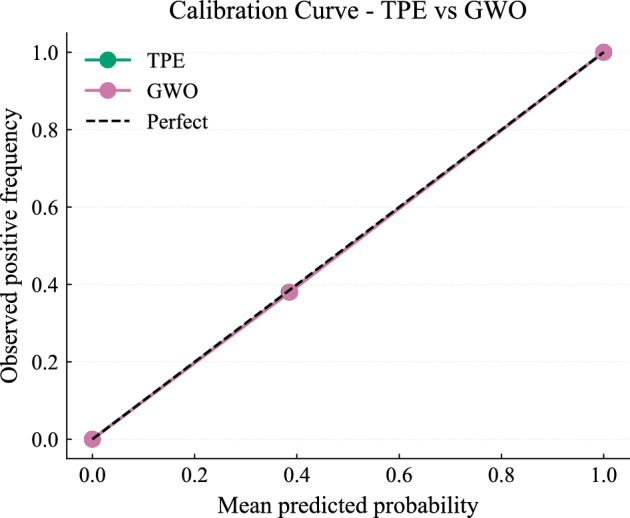
Calibration curves for TPE and GWO models..

The reliability of predicted probabilities is illustrated in Figs. 18 and 19. Both models assign high confidence to correct predictions, and their calibration curves closely follow the ideal diagonal, indicating well-calibrated outputs suitable for risk-aware decision-making.

**Fig. 20:**
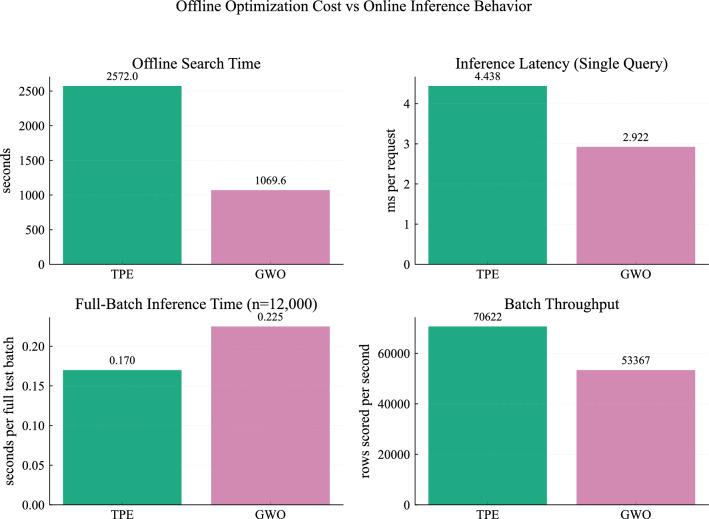
Comparison of optimization time and inference performance.

While predictive performance is nearly identical, differences emerge when considering computational efficiency. Figure 20 compares optimization cost and inference behavior.

The GWO-based model requires significantly less optimization time (1069.6 seconds compared to 2572.0 seconds for TPE). In relative terms, GWO required approximately 58% less optimization time compared to TPE. This difference can be attributed to the search behavior of the two optimization strategies. GWO explores multiple candidate solutions simultaneously through its population-based mechanism, which allows faster convergence toward suitable regions of the search space. In contrast, TPE evaluates configurations sequentially based on a probabilistic model, which can require more iterations to reach similarly effective solutions. It also achieves lower single-sample inference latency, making it more suitable for real-time applications.

In contrast, the TPE-based model achieves higher batch throughput, making it more suitable for large-scale or offline processing scenarios. This reflects a practical trade-off between different deployment scenarios.

It should be noted that the faster optimization time observed for GWO is partly influenced by repeated evaluations during early iterations, which reduces the diversity of explored configurations.

Overall, both models demonstrate highly reliable performance with extremely low error rates and well-calibrated outputs. From a SG perspective, this translates into dependable stability prediction with minimal risk of incorrect operational decisions. In practice, this means that the model can support timely interventions in unstable conditions while avoiding unnecessary control actions in stable scenarios. The choice between TPE and GWO is therefore primarily driven by deployment requirements, with TPE offering slightly stronger predictive performance and GWO providing a more efficient solution for real-time applications.

### Explainable AI results

To further assess the reliability and interpretability of the learned decision process, XAI techniques were applied to both optimized models. A comparative analysis of SHAP feature rankings and dependence patterns revealed highly consistent interpretability outcomes across TPE and GWO, with the same dominant features and closely aligned functional relationships.

These observations suggest that, although the choice of HPO strategy influences predictive performance and computational efficiency, the underlying decision structure learned by the model remains stable. In particular, both optimization approaches converge to structurally similar representations of the data, capturing the same key relationships governing system stability.

Based on this consistency, the GWO-optimized model is used for detailed interpretability analysis, as it provides a more computationally efficient configuration while preserving the same explanatory behavior.

#### Global feature importance (SHAP summary)

**Fig. 21:**
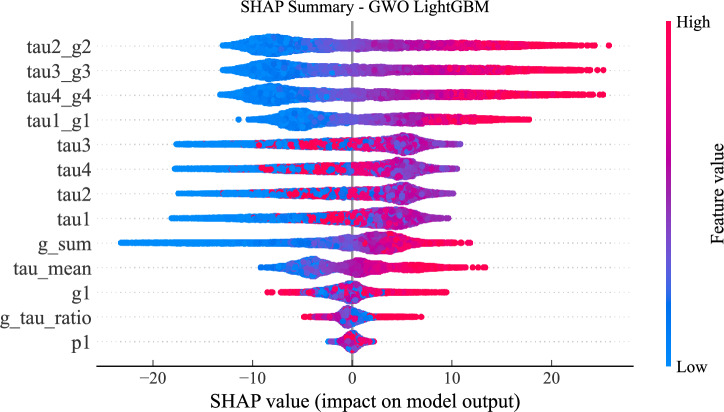
SHAP summary.

The SHAP summary plot (Fig. 21) provides a global view of feature importance across the test set. Each point represents a single prediction, where the horizontal axis indicates the contribution of a feature toward stability (positive values) or instability (negative values), and the color encodes the feature magnitude.

The results reveal a clear hierarchy, with the engineered interaction terms ($\tau _i g_i$) consistently dominating the model’s decision-making process. These features are constructed by combining reaction time ($\tau _i$), representing response delay, and price elasticity ($g_i$), representing the strength of corrective actions.

This transformation makes the coupling between delay and corrective response more explicit to the model, allowing it to capture relationships that are less evident when considering the original variables independently. High values of these interaction terms push predictions toward stability, while low values shift them toward instability. Although $\tau _i$ and $g_i$ are not direct physical measures, their interaction encodes responsiveness characteristics related to stability dynamics, demonstrating that the proposed feature engineering introduces meaningful structure without relying on explicit physical modeling.

#### SHAP dependence

**Fig. 22:**
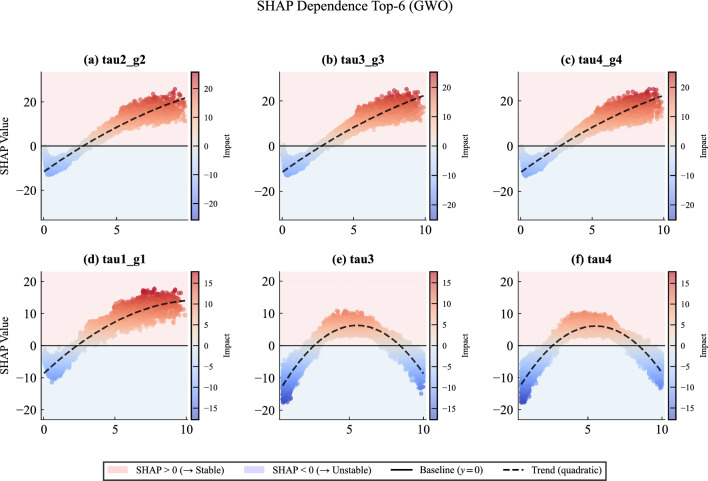
SHAP dependence plots.

The SHAP dependence plots (Fig. 22) provide a detailed view of how the most influential features affect the model output across their value ranges.

The composite features ($\tau _i g_i$ products) exhibit clear monotonic relationships, where increasing values consistently lead to higher contributions toward stability. These features combine reaction delay ($\tau _i$) and corrective strength ($g_i$), allowing the model to capture how coordinated response mechanisms influence system behavior rather than relying on either variable in isolation. As these interaction terms increase, SHAP values transition from negative to positive, indicating a shift from instability toward stability and confirming that stronger corrective responses relative to delay are associated with more stable operating conditions.

In contrast, the standalone reaction time features ($\tau _3$, $\tau _4$) display nonlinear, inverted U-shaped patterns. This indicates that both very low and very high response delays can reduce stability, while intermediate values correspond to more favorable operating conditions. SHAP values peak at mid-range values and decrease toward both extremes, showing that delay alone does not have a uniform effect on stability.

Unlike the interaction terms, these variables reflect only the timing aspect of the response, which explains their context-dependent behavior. This contrast shows that the model distinguishes between isolated delay effects and coupled interactions.

These dependence curves also reveal critical operating regions. The color gradient (red for higher values, blue for lower values) highlights where feature contributions become most influential. For the composite features, the strongest impact occurs at higher values, while for the reaction time features, the most sensitive region lies around intermediate values where stability contribution is maximized.

From an operational perspective, these results suggest that stability is not achieved by minimizing delay alone, but by maintaining an effective balance between response timing and corrective action strength, providing a more nuanced interpretation of system behavior than considering each variable independently.

#### SHAP waterfall

**Fig. 23:**
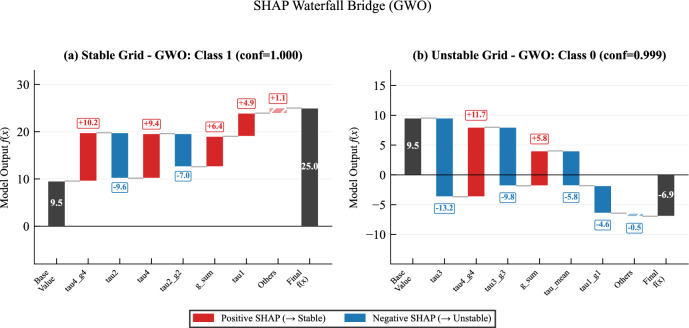
SHAP waterfall plots.

As described in Eq. 13, the waterfall charts illustrate how individual features contribute to the final prediction for specific instances. For the stable grid example (Fig. 23(a)), the model starts from a baseline value of 9.5. The prediction is primarily driven by strong positive contributions from several features, including $\tau _4 g_4$ (+ 10.2), $\tau _4$ (+ 9.4), $g_{\text {sum}}$ (+ 6.4), and $\tau _1$ (+ 4.9). These contributions outweigh negative effects from $\tau _2$ (− 9.6) and $\tau _2 g_2$ (− 7.0), resulting in a final output of approximately $f(x) = 25.0$, corresponding to a stable classification. For the unstable grid example (Fig. 23b), the model again starts from the same baseline (9.5), but the prediction is dominated by negative contributions. Although $\tau _4 g_4$ still contributes positively (+ 11.7), this effect is offset by strong negative contributions from $\tau _3$ (− 13.2), $\tau _3 g_3$ (− 9.8), $\tau _{\text {mean}}$ (− 5.8), and $\tau _1 g_1$ (− 4.6). The combined effect leads to a final output of approximately $f(x) = -6.9$, indicating an unstable state.

These examples demonstrate that the model does not rely on individual features in isolation, but rather on the balance of positive and negative contributions across multiple variables. The same feature can contribute differently depending on the overall system context, highlighting the importance of feature interactions in the decision process.

Importantly, the dominant contributions are associated with both interaction features ($\tau _i g_i$) and aggregated descriptors, indicating that stability is determined by combined delay–response behavior rather than any single variable. This supports the role of the engineered features in capturing meaningful relationships within the data.

#### ICE and partial dependence analysis

**Fig. 24:**
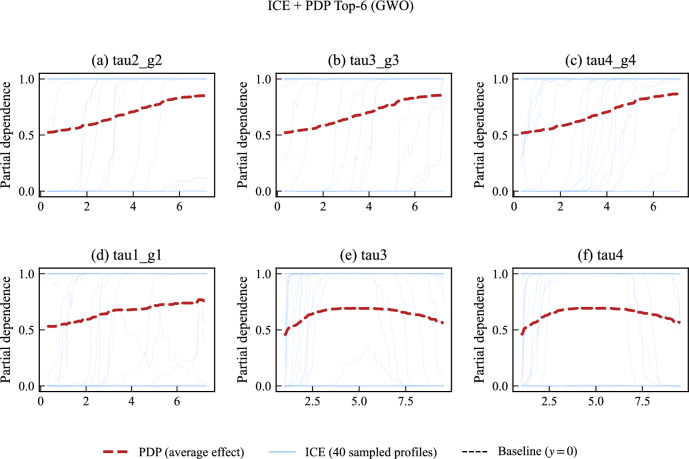
ICE/PDP analysis.

Figure 24 presents both methods for the six most influential features. The red line shows the average effect across all test instances (PDP), while the light blue lines show how predictions change for 40 randomly sampled individual instances (ICE). The black dashed baseline marks the neutral point where a feature neither promotes nor inhibits stability.

The interaction features ($\tau _2 g_2$, $\tau _3 g_3$, $\tau _4 g_4$) exhibit strong, monotonically increasing relationships. As these features increase, the predicted stability rises from approximately 0.50 at lower values to about 0.85–0.88 at higher values. The ICE curves largely follow the same upward trend, indicating that this effect is consistent across sampled instances rather than being driven by a limited subset of cases.

The feature $\tau _1 g_1$ shows a similar but weaker pattern, with a more gradual increase from approximately 0.53 to around 0.74–0.76, followed by a mild saturation at higher values. This suggests a positive but less dominant contribution compared to the other interaction terms.

The standalone reaction time features ($\tau _3$ and $\tau _4$) reveal a different behavior. Both exhibit inverted U-shaped PDP curves, where stability contributions peak at intermediate values and decrease toward both lower and higher extremes. This indicates an optimal operating range, where reaction times that are neither too fast nor too slow maximize stability. In contrast to the interaction features, the ICE curves for these variables show greater dispersion, highlighting that their effects are more sensitive to the surrounding system context.

This contrast between interaction and standalone features indicates that the model distinguishes between coupled delay–response effects and isolated timing effects, with the former providing more stable and consistent contributions to the prediction.

Overall, the ICE/PDP analysis confirms that interaction features provide consistent contributions across instances, while standalone delay variables exhibit more nonlinear and context-dependent effects.

#### Model interpretation consistency

Across the SHAP summary, dependence, waterfall, and ICE/PDP analyses, a consistent interpretation of the model behavior emerges. The results show that the model relies primarily on the engineered interaction features ($\tau _i g_i$), which capture the key interaction between response delay and corrective behavior.

These features consistently exhibit strong and stable effects across different analysis methods. The SHAP summary identifies them as the most influential variables, the dependence plots reveal clear monotonic relationships, and the ICE/PDP analysis confirms that these effects are largely consistent across sampled instances. At the same time, the waterfall plots show that the final prediction depends on the balance of multiple feature contributions rather than any single variable.

In contrast, the standalone reaction time variables exhibit nonlinear and more context-dependent behavior, indicating that their influence varies depending on the broader system configuration.

Overall, these observations suggest that the model captures structured relationships within the data that are consistently reflected across multiple interpretability techniques. This convergence of evidence increases confidence that the model is learning meaningful patterns rather than relying on spurious correlations, while remaining consistent with the physics-inspired nature of the feature representation rather than implying a direct physical model.

### Comparative with previous studies

Our research enhances the current landscape of SG stability prediction by addressing gaps in accuracy, computational efficiency, and model transparency. A comprehensive comparison of our results against previous studies is presented in Table 14.

**Table 14:** Comparison with previous studies on SG stability prediction.

Study	Model architecture	Dataset	Acc. (%)	HPO Strategy	XAI framework
Tiwari et al.^23^	SVM	Augmented	98.00	None	None
Ibrar et al.^59^	XGBoost + ROS	Original	96.80	None	None
Mostafa et al.^60^	Penalized LR	Augmented	96.00	None	None
Breviglieri et al.^30^	DL (Nadam)	Augmented	99.62	Manual	None
Alsirhani et al.^61^	MLP-ELM	Original	95.80	None	None
Franović et al.^62^	XGBoost	Augmented	99.70	Grid Search	None
Mohsen et al.^11^	ANN	Augmented	97.36	Manual	None
Ucar et al.^33^	GBM	Both	99.60	AutoML (H2O)	SHAP + PDP
Allal et al.^34^	CatBoost + K-SMOTE	Augmented	99.60	None	SHAP + LIME
Hassan et al.^24^	Stacked Ensemble	Augmented	97.77	GridSearchCV	None
Lakshmanarao et al.^27^	ANN + SVM Fusion	Original	98.90	None	None
Alaerjan et al.^25^	Voting (RF+XGB+GB)	Augmented	99.80	None	None
Ahakonye et al.^26^	1D CNN	Augmented	99.79	Manual	None
Karim et al.^32^	Guide-WWPA + LSTM	Original	99.70	Guide-WWPA	None
Cifci et al.^35^	ANN	Original	96.20	None	SHAP + ICE
Wang et al.^12^	BO-SVM	Original	96.00	BO vs. Grid	SHAP
This study	LightGBM	Augmented	99.90–99.95	GWO & TPE	SHAP ,Waterfall, PDP, ICE

While our GWO-LightGBM framework achieves a state-of-the-art performance with an accuracy of 99.90%, the primary value of this work lies in the integration of its three core components.

First, the findings show that the proposed feature engineering approach is a key factor underlying the high performance. By introducing interaction-based features that capture the relationship between response delay and corrective action, the model achieves improved performance compared to studies relying on raw features and complex architectures, such as the ANN approach by Cifci et al.^35^. This suggests that, for structured systems such as SGs, carefully designed feature representations can be more effective than increasing model complexity alone.

Second, the comparative performance of our HPO strategies offers evidence-based guidance on computational efficiency. While previous works like Wang et al.^12^ focused on comparing basic GridSearch to Bayesian methods, our simultaneous evaluation of TPE and GWO shows that metaheuristic approaches can achieve comparable predictive performance while reducing computational overhead by approximately 58%. This efficiency is a critical factor for real-time grid applications where periodic model retraining is necessary.

Third, the multi-layered XAI framework provides a level of validation that is largely absent in the existing literature. Most studies cluster between 99.6% and 99.8%, yet few provide the interpretability required for safety-critical deployment. By combining global explanations (SHAP) with instance-level analysis (ICE and PDP), the proposed framework provides consistent and interpretable patterns that align with expected system behavior, effectively bridging the gap between “black-box” ML and trustworthy grid operations.

## Conclusion, limitations, future works

This study presents a unified ML framework for SG stability prediction, combining feature engineering, HPO, and XAI within a consistent pipeline. A diverse set of models was systematically evaluated, with LightGBM emerging as the most effective approach under both TPE and GWO optimization strategies. Model performance was assessed using multiple evaluation metrics, including accuracy, precision, recall, F1-score, AUC, log loss, Cohen’s kappa, and MCC.

The results highlight several important insights. Carefully designed feature representations play a central role in achieving high predictive performance, emphasizing the value of domain-informed feature construction. The comparison between TPE and GWO shows that both approaches achieve comparable predictive performance while differing in computational efficiency, providing flexibility depending on deployment requirements. In addition, the integration of multiple XAI techniques, including SHAP, ICE, and PDP, provides consistent and complementary explanations of the model’s behavior, supporting the interpretability of the proposed framework.

We applied feature engineering in this study to obtain higher accuracy. Instead of relying solely on raw measurements, a set of physics-inspired features was constructed to better capture relationships within the data. The coupling terms ($\tau _i \times g_j$), aggregate statistics ($\tau _{mean}$, $g_{sum}$), and ratio-based features ($g_{tau\_ratio}$) improved class separation in the feature space. The final optimized models achieved near-perfect predictive performance on the 60,000-sample dataset, with the TPE-based LightGBM reaching 99.95% accuracy and the GWO-based LightGBM reaching 99.90%. This result highlights the importance of domain-informed feature design alongside model selection. The XAI analysis indicates that the model relies on consistent and structured patterns associated with these engineered features, rather than isolated or unstable effects.

We compared the TPE with GWO for HPO. Both methods achieve comparable predictive performance, while GWO reduces the optimization time by approximately 58%, offering a more efficient alternative for scenarios requiring periodic model updates. This is practical for operators who need to retune models periodically. Wang et al.^12^ compared GridSearchCV with Bayesian optimization, but we advanced this by comparing two advanced strategies on the same dataset.

Our XAI system integrates four methodologies: SHAP summary charts, SHAP dependence plots, SHAP force plots, and ICE plots. This multi-method approach provided consistent evidence of structured relationships in the data. The ICE plots were very close together, which suggests that the predictions are consistent across the dataset. This is important for grid operators who need trustworthy performance.

We show the ML requires three elements: domain expertise in feature engineering, systematic methodology in optimization, and rigorous validation through interpretability. When these three combine, models can achieve very high predictive performance while maintaining consistent and interpretable behavior.

The way forward for ML in critical infrastructure is clear: use deep domain knowledge, a methodical approach, and openness. This paper outlines a framework for constructing reliable ML systems in which operator trust and regulatory compliance are primary design objectives rather than secondary considerations.

The results indicate that ML-based approaches can effectively support SG stability monitoring by enabling accurate and data-driven decision-making, contributing to more reliable and efficient grid operation. Nevertheless, this study was restricted to a controlled dataset with homogeneous input and idealized settings, which may limit the generalizability of the findings to real-world grid systems. Model performance may be impacted in reality by problems including sensor noise, missing data, and dynamic operating conditions.

A limitation of this study is that the proposed framework was evaluated on a single dataset, which may limit its ability to generalize to different data distributions. In real-world SG environments, data characteristics can vary due to differences in grid topology, measurement processes, and operating conditions. Therefore, the reported performance may not fully reflect behavior under more complex or heterogeneous scenarios.

Future work should focus on evaluating the proposed framework on multiple and more diverse datasets, including real-world SG data, to better assess its generalization capability. In addition, extending the preprocessing pipeline to different ML and DL models may provide further insights into its adaptability. Other important directions include handling noisy and incomplete data, validating the approach in real-time SG environments, and exploring hybrid models that combine ML techniques with physical modeling principles.

SGs can also be vulnerable to cyber-attacks, which were not considered in this study. Incorporating security-related scenarios and robustness mechanisms represents an important direction for future research, particularly for practical deployment in real-world systems.

Beyond predictive performance and interpretability, stability predictions may support technical grid operation and decision-making processes. In addition, their potential impact on energy market analysis represents an interesting direction for future investigation.

## Data Availability

The dataset analysed during this study is publicly available in the Kaggle repository (“Smart grid stability dataset”) at https://www.kaggle.com/datasets/pcbreviglieri/smart-grid-stability. The dataset is openly accessible without restriction under Kaggle’s data usage terms. All preprocessing procedures applied to the data are fully described in the manuscript. No additional datasets were generated.

## List of symbols

$\tau _i$: Rotor angle deviation of gen *i*
$g_i$: Damping coefficient of gen *i*
$p_i$: Power output of gen *i*
*f*(*x*): Model output (prediction)
*E*[*f*(*x*)]: Expected value
$\phi _i(x)$: SHAP value for feature *i*
*n*: Total number of features
$\alpha$: Alpha wolf (best in GWO)
$\beta$: Beta wolf (2nd best in GWO)
$\delta$: Delta wolf (3rd best in GWO)
$\omega$: Omega wolf (rest of GWO)
*M*: Inertia constant
*D*: Damping coefficient
$P_m$: Mechanical power input
$P_e$: Electrical power output

## Abbreviations

AI: Artificial intelligence
ANN: Artificial neural networks
ANOVA: Analysis of variance
AUC: Area under the curve
BO: Bayesian optimization
CatBoost: Categorical boosting
CNN: Convolutional neural networks
CV: Cross-validation
CI: Confidence interval
DL: Deep learning
EDA: Exploratory data analysis
EI: Expected improvement
F1: F1-score
FN: False negatives
FP: False positives
GB: GradientBoosting
GWO: Grey wolf optimizer
HPO: Hyperparameter optimization
ICE: Individual cond. expectation
KNN: K-nearest neighbors
LDA: Linear discriminant analysis
LightGBM: Light gradient boosting
LIME: Local interpretable exp.
LSTM: Long short-term memory
MCC: Matthews correlation coeff.
ML: Machine learning
PDP: Partial dependence plot
QDA: Quadratic discriminant analysis
RF: Random forest
RFE: Recursive feature elimination
ROC: Receiver operating char.
ROS: Random over-sampling
SAMME: Stagewise additive modeling
SG: Smart grid
SGD: Stochastic gradient descent
SHAP: SHapley Additive exPlanations
SMOTE: Synthetic minority over-sampling
SVM: Support vector machine
TP: True positives
TN: True negatives
TPE: Tree-structured Parzen est.
UCI: Univ. of California, Irvine
WWPA: Water wave particle alg.
XAI: Explainable AI
XGBoost: Extreme gradient boosting

## Code availability

All code used in this study (data preprocessing, feature engineering, model training, HPO, evaluation, and XAI analysis) is available in a public GitHub repository: https://github.com/Omar-Zantot/smart-grid-stability-v01. The code is permanently archived with a DOI for long-term access and citation: https://zenodo.org/records/19263058. The repository includes Jupyter notebooks for all analysis stages, Python scripts, configuration files with hyperparameters and random seeds, a requirements file specifying all dependencies, and a README with reproduction instructions. The code is provided under the MIT License.
